# A transport and retention mechanism for the sustained distal localization of Spn-F–IKKε during *Drosophila* bristle elongation

**DOI:** 10.1242/dev.121863

**Published:** 2015-07-01

**Authors:** Tetsuhisa Otani, Kenzi Oshima, Akiyo Kimpara, Michiko Takeda, Uri Abdu, Shigeo Hayashi

**Affiliations:** 1 Laboratory for Morphogenetic Signaling, RIKEN Center for Developmental Biology, Kobe, Hyogo 650-0047, Japan; 2 Department of Life Sciences andthe National Institute for Biotechnology in the Negev, Ben-Gurion University, Beer-Sheva 84105, Israel; 3 Department of Biology, Kobe University Graduate School of Science, Kobe, Hyogo 657-8501, Japan

**Keywords:** Cell elongation, Cell polarity, IKKε, Ik2, Dynein, *Drosophila*

## Abstract

Stable localization of the signaling complex is essential for the robust morphogenesis of polarized cells. Cell elongation involves molecular signaling centers that coordinately regulate intracellular transport and cytoskeletal structures. In *Drosophila* bristle elongation, the protein kinase IKKε is activated at the distal tip of the growing bristle and regulates the shuttling movement of recycling endosomes and cytoskeletal organization. However, how the distal tip localization of IKKε is established and maintained during bristle elongation is unknown. Here, we demonstrate that IKKε distal tip localization is regulated by Spindle-F (Spn-F), which is stably retained at the distal tip and functions as an adaptor linking IKKε to cytoplasmic dynein. We found that Javelin-like (Jvl) is a key regulator of Spn-F retention. In *jvl* mutant bristles, IKKε and Spn-F initially localize to the distal tip but fail to be retained there. In S2 cells, particles that stain positively for Jvl or Spn-F move in a microtubule-dependent manner, whereas Jvl and Spn-F double-positive particles are immobile, indicating that Jvl and Spn-F are transported separately and, upon forming a complex, immobilize each other. These results suggest that polarized transport and selective retention regulate the distal tip localization of the Spn-F–IKKε complex during bristle cell elongation.

## Abstract (precis)

**Summary:** In the *Drosophila* bristle, the microtubule binding protein Jvl, the adaptor Spn-F and cytoplasmic dynein are required for localised transport and retention of polarised signalling factors.

## INTRODUCTION

Highly polarized cells, such as neurons and epithelial cells, rely heavily on intracellular transport mechanisms for their functional differentiation (Apodaca et al., 2012; Maeder et al., 2014). Disrupted intracellular transport systems lead to a variety of diseases, including neurodegeneration and microvillus inclusion diseases (Encalada and Goldstein, 2014; Millecamps and Julien, 2013; Golachowska et al., 2010). Accurate intracellular transport is ensured by the polarized cytoskeleton and by the adaptor protein-mediated recognition of specific cargoes by molecular motors (Maeder et al., 2014; Franker and Hoogenraad, 2013). Molecular motors play central roles in intracellular transport, and have diversified through evolution (Vale, 2003). However, the diversity of molecular motors is not sufficient to explain transport specificity, as various cargoes often share the same motor while being transported to distinct locations. For example, cytoplasmic dynein is the major microtubule minus-end motor and transports a variety of cargoes including the Golgi apparatus, endosomes and RNAs (Kardon and Vale, 2009). Evidence suggests that the fate of cargo is determined not only by cargo-motor recognition, which occurs upon cargo loading, but also at the cargo destination site. For instance, in axonal transport some cargoes, such as dense core vesicles and synaptic vesicles, are inefficiently captured at synaptic boutons and circulate within the axon (Wong et al., 2012), whereas others, such as mitochondria, are stably retained at synapses (Kang et al., 2008). Although the precise regulation of cargo transport is important for the functional differentiation of various polarized cells, the underlying molecular mechanisms remain poorly understood.

Cell elongation is a widely observed morphogenetic event that requires the coordinated input of intracellular transport, the cytoskeleton and cell polarity (Hepler et al., 2001; Riquelme, 2013). *Drosophila* bristles, which are hair-like unicellular structures that function as external sensory organs, are formed by the elongation of trichogen cells, which can grow up to 350 µm in 1 day during the pupal stage (Lees and Picken, 1945; Lees and Waddington, 1942; Tilney and DeRosier, 2005). IκB kinase ε [IKKε; also known as IκB kinase-like 2 (Ik2)] acts at the distal tip of growing bristles and functions as a signaling center to regulate the bidirectional shuttling of Rab11-positive recycling endosomes during bristle elongation (Otani et al., 2011). Rab11-positive vesicles are transported to the distal tip by interacting with cytoplasmic dynein via an adaptor protein Nuf/Rab11FIP3 (Otani et al., 2011; Riggs et al., 2007). At the distal tip, IKKε phosphorylates Nuf to inactivate dynein-dependent trafficking, thereby promoting the directional switching of the recycling endosomes (Gould, 2011; Otani et al., 2011). In addition to its role in endosome trafficking, IKKε regulates the organization of both actin and microtubules (Otani et al., 2011; Bitan et al., 2010, 2012). However, how IKKε is localized to the distal tip of growing bristles is unknown.

Spindle-F (Spn-F) is a coiled-coil protein that interacts with IKKε and has been implicated in regulating IKKε polarized activation (Abdu et al., 2006; Dubin-Bar et al., 2008). In oocytes, the intracellular localizations of Spn-F and IKKε depend on each other (Dubin-Bar et al., 2008), and *spn-F* and *ikkε* mutants show similar bristle morphology and oocyte polarization phenotypes, suggesting that they function together (Abdu et al., 2006; Koto et al., 2009; Oshima et al., 2006; Otani et al., 2011; Shapiro and Anderson, 2006). Several proteins other than IKKε, including Cut up (Ctp)/dynein light chain (LC8) and Javelin-like (Jvl), are reported to interact with Spn-F (Abdu et al., 2006; Dubin-Bar et al., 2011). It was proposed that Spn-F interacts with cytoplasmic dynein via Ctp to localize the Spn-F–IKKε complex to microtubule minus ends (Abdu et al., 2006). However, subsequent structural studies indicated that Ctp/LC8 cannot simultaneously bind dynein and cargo molecules, challenging this model (Benison et al., 2007; Rapali et al., 2011; Williams et al., 2007). On the other hand, IKKε can phosphorylate Spn-F, suggesting that Spn-F might act downstream of IKKε (Dubin-Bar et al., 2008). Interestingly, another Spn-F-interacting protein, Jvl, was recently shown to regulate the polarized activation of IKKε in oocytes (Amsalem et al., 2013). Although Jvl can interact with microtubules (Dubin-Bar et al., 2011), how it regulates the polarized activation of IKKε is unknown.

In this study, we sought to understand how IKKε, Spn-F and Jvl interact with each other to establish and maintain the signaling center during bristle elongation.

## RESULTS

### Spn-F stably localizes to the distal tip of the elongating bristle

**Fig. 1.**
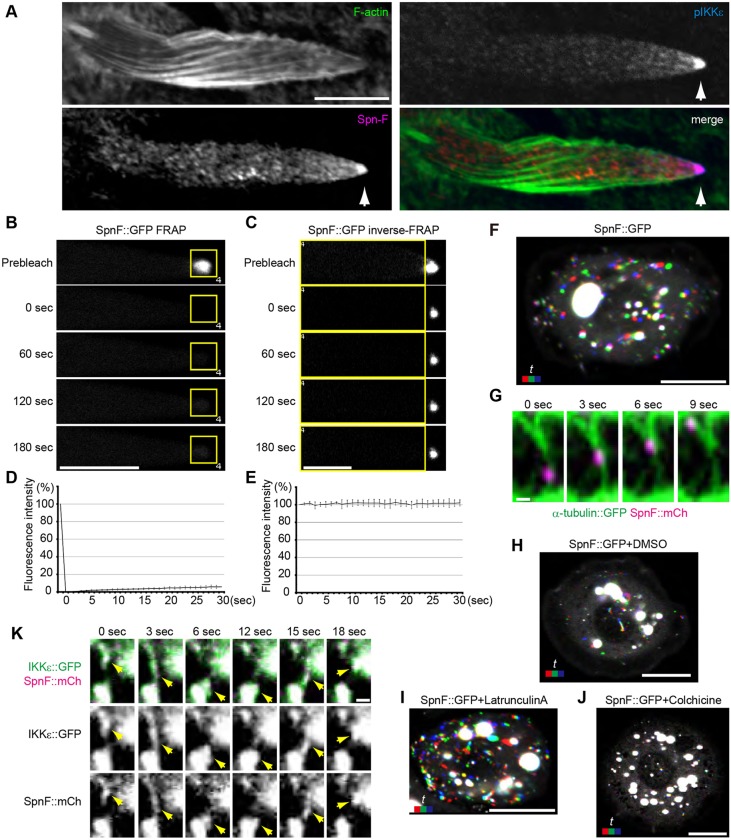
**Spn-F stably localizes to the distal tip during *Drosophila* bristle elongation.** (A) Localization of F-actin (green), pIKKε (blue) and Spn-F (red) in bristles 33 h after puparium formation (APF). pIKKε and Spn-F signals colocalize at the distal tip (arrowheads). (B,C) FRAP (B) and inverse-FRAP (C) analyses of SpnF::GFP at 33 h APF. Yellow boxes indicate the photobleached region. (D,E) Quantitation of FRAP (D) and inverse-FRAP (E) results. Error bars indicate s.d.; *n*=3-4 bristles analyzed.(F-J) Spn-F mobility in S2 cells. (F) SpnF::GFP particles move in S2 cells. (G) SpnF::mCh (magenta) particles move along microtubules (green). (H-J) SpnF::GFP movement depends on microtubules. S2 cells expressing SpnF::GFP were treated with DMSO (H), 1 µM Latrunculin A (I) or 10 µM Colchicine (J). (K) IKKε::GFP (green) and Spn-F::mCh (magenta) move together in S2 cells (arrowheads). (F,H-J) Red, 0 s; green, 3 s; blue, 6 s. See also supplementary material Movies 1-8. Scale bars: 10 µm in A-C,F,H-J; 1 µm in G,K.

To elucidate the relationship between IKKε and Spn-F in bristle elongation, we co-stained developing bristles with anti-Spn-F and anti-phosphorylated IKKε (at serine 175; pIKKε) antibodies. Spn-F and pIKKε accumulated and colocalized at the tip of growing bristles (Fig. 1A) (Bitan et al., 2010; Otani et al., 2011).

**Fig. 2.**
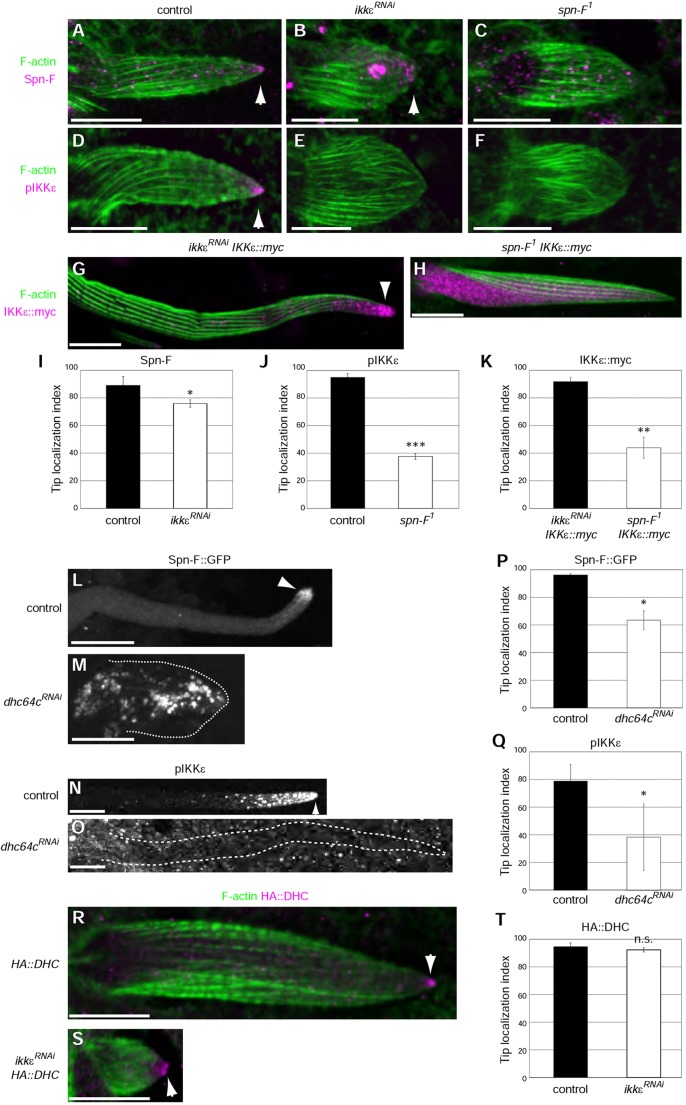
**Spn-F and cytoplasmic dynein are required for the polarized localization of IKKε.** (A-F) Spn-F (A-C, magenta) and pIKKε (D-F, magenta) localization in control, *ikkε^RNAi^* and *spn-F^1^* mutant bristles at 33 h APF. Spn-F localizes to the tip of control (A) and *ikkε^RNAi^* (B) bristles (arrowhead). Some aggregation of Spn-F in the bristle shaft is observed in *ikkε^RNAi^* bristles. (C) The Spn-F signal is greatly reduced in *spn-F^1^* bristles. The remaining signals were background, as *spn-F^1^* is a null allele. (D) pIKKε localizes to the tip of control bristles (arrowhead). (E) The pIKKε signal is lost in *ikkε^RNAi^* bristles. (F) pIKKε tip localization is lost in *spn-F^1^* bristles. (G,H) IKKε::myc (magenta) localizes to the distal tip (arrowhead) at 40 h APF in *ikkε^RNAi^ IKKε::myc* (G) but not in *spn-F^1^* (H) bristles. Bristle morphology is shown by F-actin (green) in A-H. (I-K) Quantification of Spn-F (I), pIKKε (J) and IKKε::myc (K) tip localization. (L-O) Spn-F::GFP (L,M) and pIKKε (N,O) localization in control and *Dhc64C* RNAi (*Dhc64C^RNAi^*) bristles. Spn-F::GFP and pIKKε localize to the tip (arrowheads) of control bristles (36 h APF in L, 40 h APF in N) but not in *Dhc64C^RNAi^* bristles (20 h APF in M, 24 h APF in O, animals raised at 32°C). Dotted lines (M,O) outline the cell. (P,Q) Quantification of the tip localization of SpnF::GFP (P) and pIKKε (Q). (R,S) HA::DHC localizes to the tip (arrowheads) of control (R) and *ikkε^RNAi^* (S) bristles at 33 h APF. (T) Quantification of HA::DHC tip localization. Error bars indicate s.d. *P<0.05, **P<0.005, ***P<0.0005; n.s., not significant. See also supplementary material Figs S1 and S2. Scale bars: 10 µm.

**Fig. 3.**
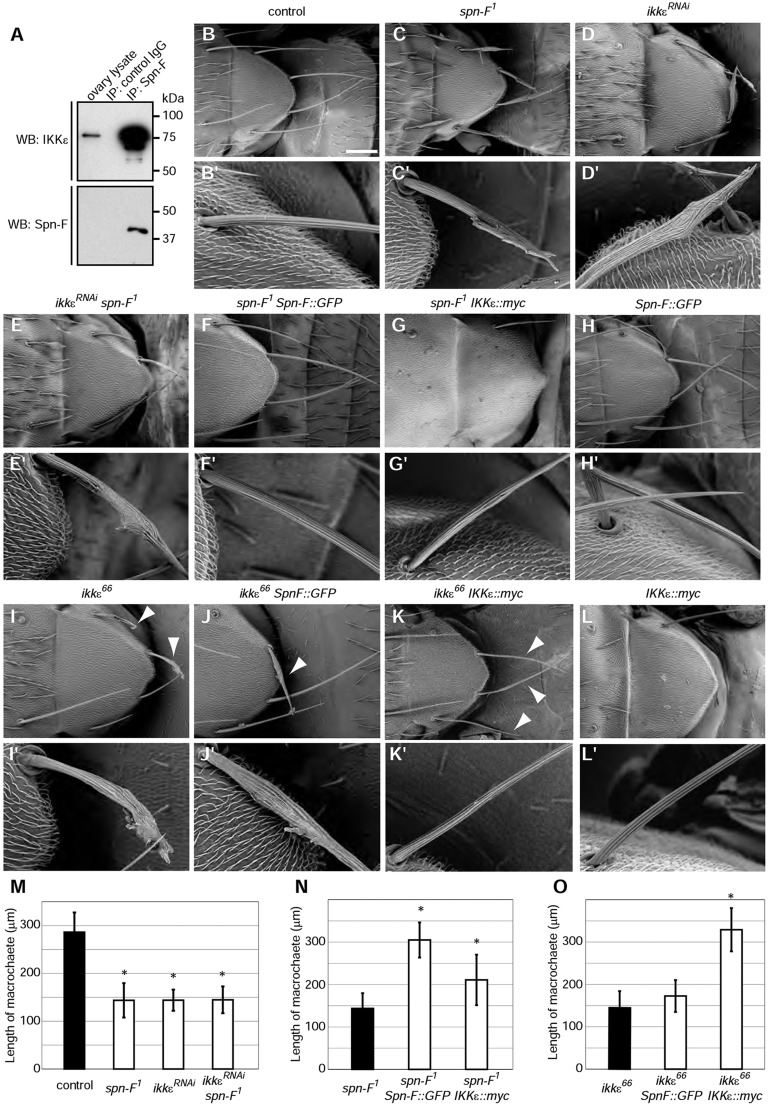
***ikkε* is epistatic to *spn-F*.** (A) Co-immunoprecipitation of IKKε with Spn-F. The Spn-F signal in the ovary lysate is too weak to detect. IP, immunoprecipitation; WB, western blot. (B-L) SEM images of scutellar bristles of the indicated genotypes. (B′-L′) Magnified images of bristle morphology. (B) Control bristles. *spn-F^1^* (C), *ikkε^RNAi^* (D) and *ikkε^RNAi^ spn-F^1^* (E) bristles are short and branched. (F) Spn-F overexpression rescues the *spn-F^1^* mutant bristle phenotype. (G) IKKε overexpression reduces the number of sensory organs and suppresses the *spn-F^1^* bristle phenotype. (H) Spn-F overexpression does not severely affect bristle morphology. (I) *ikkε^66^* mutant bristles are short and branched (arrowheads). (J) Spn-F overexpression does not suppress the *ikkε^66^* bristle phenotype (arrowhead). (K) IKKε overexpression rescues the *ikkε^66^* bristle phenotype (arrowheads). (L) IKKε overexpression reduces the number of sensory organs, although the remaining bristles are morphologically normal. (M-O) Quantification of bristle morphology. Length of scutellar bristles was measured. Error bars indicate s.d.; *n*>3 bristles analyzed. *P<0.05. See also supplementary material Fig. S2. Scale bar: 100 µm.

We next examined the dynamics of Spn-F by expressing functional Spn-F::GFP, which accumulated at the distal tip (Fig. 2L) and could rescue the *spn-F^1^* mutant bristle morphology phenotype (Fig. 3F,N). Fluorescence recovery after photobleaching (FRAP) and inverse-FRAP experiments revealed that the Spn-F::GFP at the distal tip did not turnover within 3 min (Fig. 1B-E; supplementary material Movies 1 and 2), as distinct from GFP::Rab11, which turned over within 30 s (Otani et al., 2011). These results indicate that the distal tip localization of Spn-F is stable.

### Spn-F moves along microtubules in *Drosophila* S2 cells

To study the Spn-F transport mechanism, we expressed Spn-F::GFP in cultured Schneider 2 (S2) cells and observed its motion. In S2 cells, Spn-F::GFP was localized to puncta that moved along microtubules (Fig. 1F,G; supplementary material Movies 3 and 4). This motion still occurred upon treatment with DMSO (Fig. 1H; supplementary material Movie 5) or the actin-depolymerizing drug Latrunculin A (Fig. 1I; supplementary material Movie 6). However, the microtubule-depolymerizing drug Colchicine abolished Spn-F::GFP movement, suggesting that it requires microtubules (Fig. 1J; supplementary material Movie 7). Consistent with the interaction between Spn-F and IKKε (Dubin-Bar et al., 2008), we found that IKKε::GFP and Spn-F::mCh colocalized and moved together in S2 cells (Fig. 1K; supplementary material Movie 8). These results indicated that Spn-F and IKKε are transported together along microtubules in S2 cells.

### Dynein and Spn-F are required for the polarized localization of IKKε

We next examined how the distal tip localization of Spn-F and IKKε is regulated in growing bristles. In control bristles, both Spn-F and pIKKε localized to the distal tip (Fig. 2A,D). In *ikkε^RNAi^* bristles, a subset of Spn-F localized to the distal tip, although some also accumulated within the shaft (Fig. 2A-C). By contrast, the pIKKε signal did not accumulate at the distal tip in *spn-F^1^* bristles (Fig. 2D-F). The tip localization was quantitated by measuring the ‘tip index’ (supplementary material Fig. S1A,B), which has a value of 100 when the signals are completely localized to the distal tip, 0 when they are completely located within the cell body and 50 when they are diffuse (supplementary material Fig. S1C). Spn-F distal tip localization was slightly diminished in *ikkε^RNAi^* bristles (Fig. 2I), whereas pIKKε tip localization was severely disorganized in *spn-F^1^* bristles (Fig. 2J).

To monitor IKKε localization, we used IKKε::myc protein because no available anti-IKKε antibody is sensitive enough for this purpose. As IKKε overexpression is toxic to flies and causes changes in bristle cell fate or morphology (Fig. 3L) (Otani et al., 2011), we replaced some of the endogenous IKKε with low levels of epitope-tagged IKKε by coexpressing IKKε::myc with IKKε hairpin RNA, which targets both endogenous and exogenous IKKε. This resulted in normal bristle morphology (supplementary material Fig. S1D), and IKKε::myc protein accumulated at the distal tip in control bristles (Fig. 2G) but not in *spn-F^1^* bristles (Fig. 2H,K), indicating that Spn-F is required for IKKε localization to the distal tip in growing bristles.

We next focused on the microtubule minus-end motor cytoplasmic dynein, as the minus-ends of stable microtubules are oriented toward the distal tip (Bitan et al., 2010, 2012). RNAi of *Dhc64C*, the *Drosophila* cytoplasmic dynein heavy chain (Gepner et al., 1996; Li et al., 1994; Rasmusson et al., 1994), caused the mislocalization of Spn-F::GFP (Fig. 2L,M,P) and pIKKε (Fig. 2N,O,Q). HA-tagged dynein heavy chain (HA::DHC) localized to the distal tip in both control and *ikkε^RNAi^* bristles (Fig. 2R-T). These results suggested that cytoplasmic dynein is required for Spn-F tip localization, and that Spn-F is in turn required for IKKε tip localization.

### *ikkε* is epistatic to *spn-F*

To investigate the relationship between Spn-F and IKKε, we first examined their physical interaction. Spn-F and IKKε are reported to interact with each other (Dubin-Bar et al., 2008), which was confirmed by immunoprecipitation experiments in S2 cells. Overexpressed (supplementary material Fig. S2A) or endogenous (supplementary material Fig. S2B) IKKε co-precipitated with overexpressed Spn-F. Moreover, endogenous Spn-F and IKKε were co-immunoprecipitated from ovary lysates, indicating that they form a complex *in vivo* (Fig. 3A; supplementary material Fig. S2C). These results demonstrated that Spn-F and IKKε interact with each other.

We next examined the genetic interactions between *spn-F* and *ikkε* in bristle morphology. Wild-type bristles have a thin, elongated, tapered morphology (Fig. 3B), whereas in *spn-F^1^* or *ikkε^RNAi^* flies the bristles are short, branched and have a characteristic swollen region (Fig. 3C,D) (Abdu et al., 2006; Oshima et al., 2006; Shapiro and Anderson, 2006). We generated *ikkε^RNAi^ spn-F^1^* double-mutant bristles and found no additive effects as compared with the single mutants (Fig. 3E). This was confirmed by measuring the lengths of the scutellar bristles in each genotype (Fig. 3M). The lack of additive effects in *ikkε^RNAi^ spn-F^1^* double-mutant bristles suggested that *spn-F* and *ikkε* function in the same genetic pathway.

To elucidate the relationship between *spn-F* and *ikkε*, we performed a genetic epistasis analysis. The low-level expression of IKKε::myc in wild-type flies reduced the number of sensory organs (Fig. 3L), which is consistent with a previous report suggesting a role for IKKε in sensory organ precursor development (Kuranaga et al., 2006), although the morphology of the remaining bristles was normal, indicating that IKKε::myc does not affect bristle morphogenesis once the cell fate has been determined (Fig. 3L). IKKε::myc rescued the defects in *ikkε* mutant bristles (Fig. 3K,O). Furthermore, IKKε::myc suppressed the bristle morphology defects in *spn-F^1^* mutants (Fig. 3C,G,N). In some animals, bristles with a hooked morphology were occasionally observed (supplementary material Fig. S2D), suggesting that IKKε requires Spn-F to fully exert its function. By contrast, when Spn-F was overexpressed, the bristle morphology was largely normal, with only the occasional appearance of hooked bristles (Fig. 3H). Spn-F overexpression rescued the defects in *spn-F^1^* bristles (Fig. 3F,N), but failed to suppress the bristle morphology defects in *ikkε* mutants (Fig. 3I,J,O; supplementary material Fig. S2E,F). Taken together, these results demonstrated that *ikkε* is epistatic to *spn-F*.

### Spn-F interacts with IKKε, Ctp and DHC through distinct regions

**Fig. 4.**
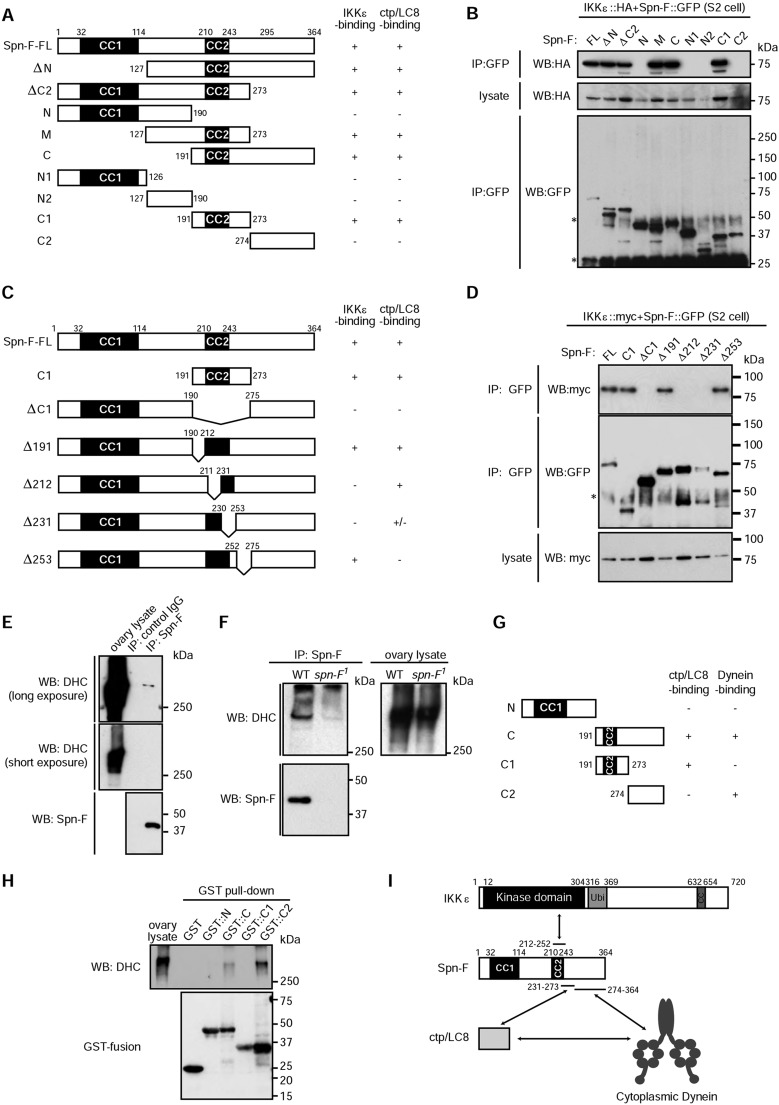
**Biochemical characterization of the IKKε–Spn-F–dynein complex.** (A) The Spn-F constructs used in B. CC1 and CC2, coiled-coil regions 1 and 2. (B) Co-immunoprecipitation analysis showing that the Spn-F C1 region is necessary and sufficient to interact with IKKε::HA. (C) The Spn-F constructs used in D. (D) Co-immunoprecipitation analysis showing that amino acids 212-252 of Spn-F are required for its interaction with IKKε::myc. (E,F) DHC co-immunoprecipitates with Spn-F. (G) The GST-fusion Spn-F constructs used in H. (H) Pull-down analysis showing that the Spn-F C2 region interacts with DHC. (I) Summary of interactions between IKKε, Spn-F, Ctp and cytoplasmic dynein. See also supplementary material Fig. S3.

The above results suggested that Spn-F acts upstream of IKKε to regulate IKKε distal tip localization. To elucidate the molecular mechanisms of this IKKε localization, we performed a structure-function analysis of Spn-F. The Spn-F protein has two coiled-coil regions (CCs), which we designated CC1 and CC2 (Fig. 4A). We generated various deletion mutants of Spn-F (Fig. 4A) and tested their ability to interact with IKKε in S2 cells, and found that the C1 construct (comprising amino acids 191-273), which contains CC2, is necessary and sufficient for Spn-F to interact with IKKε (Fig. 4B).

Ctp/LC8 has been proposed to be a cargo adaptor for Spn-F and cytoplasmic dynein (supplementary material Fig. S3A) (Abdu et al., 2006). We next determined the Ctp-interacting region of Spn-F, and found that the C1 construct was also necessary and sufficient for Spn-F to interact with Ctp (supplementary material Fig. S3B). As IKKε and Ctp both interacted with the CC2-containing region of Spn-F, we examined whether their bindings to Spn-F were mutually exclusive. However, we found that IKKε and Ctp formed a complex in an Spn-F-dependent manner (supplementary material Fig. S3C), suggesting that IKKε and Ctp interact with distinct regions of Spn-F. Further dissection of the Spn-F C1 region identified amino acids 212-252 as the IKKε-interacting region (Fig. 4C,D), whereas the Ctp-interacting region was mapped to amino acids 231-274 (supplementary material Fig. S3D). These results suggested that IKKε and Ctp interact with Spn-F at distinct but overlapping regions.

To test whether Ctp mediates the interaction between Spn-F and cytoplasmic dynein, we analyzed the interaction between Spn-F and dynein heavy chain (DHC). Immunoprecipitation using ovary lysates revealed that endogenous DHC co-precipitated with Spn-F (Fig. 4E,F), suggesting that Spn-F forms a complex with cytoplasmic dynein. To determine the cytoplasmic dynein-binding region of Spn-F, we prepared GST-fusion Spn-F fragments (Fig. 4G) and performed pull-down experiments using ovary lysates. DHC interacted with the C-terminus of Spn-F (C2 region) but not with the Ctp-interacting (C1) region (Fig. 4H), indicating that Ctp binding is dispensable for Spn-F to interact with cytoplasmic dynein. These results demonstrate that Spn-F interacts with IKKε, Ctp and cytoplasmic dynein through distinct regions (Fig. 4I).

### Spn-F acts as a cargo adaptor between IKKε and cytoplasmic dynein

**Fig. 5.**
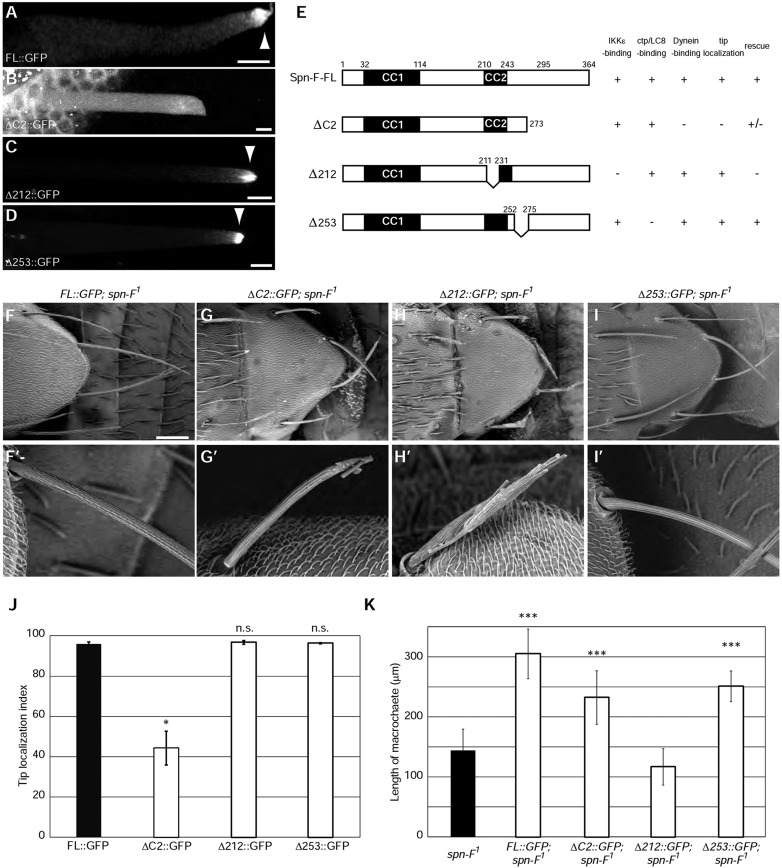
**The Spn-F dynein-interacting region and IKKε-interacting region are both required for bristle morphogenesis.** (A-D) Localization of Spn-F deletion mutants in developing bristles at 36 h APF. FL::GFP (A), Δ212::GFP (C) and Δ253::GFP (D), but not ΔC2::GFP (B), localize to the distal tip (arrowheads). (E) Characteristics of Spn-F deletion mutants. (F-I) SEM images of scutellar bristles of the indicated genotypes. (F′-I′) Magnified images of bristle morphology. FL::GFP (F′) and Δ253::GFP (I′), but not Δ212::GFP (H′), rescue the *spn-F^1^* bristle phenotype. ΔC2::GFP partially suppressed the *spn-F^1^* bristle phenotype (G′). (J) Quantification of the tip localization of Spn-F deletion mutants. *n*=2-3 bristles analyzed. (K) Quantification of bristle morphology. *n*>5 bristles analyzed. Error bars indicate s.d. *P<0.05, ***P<0.0005; n.s., not significant. See also supplementary material Fig. S4. Scale bars: 5 µm in A-D; 100 µm in F-I.

The results obtained so far suggested that Spn-F acts as a cargo adaptor to couple IKKε to cytoplasmic dynein. To test this model, we examined the ability of the Spn-F deletion mutants to localize to the tip of growing bristles in transgenic flies. We found that the dynein-interacting (C2) region of Spn-F was necessary and sufficient for its tip localization (Fig. 5A-E,J; supplementary material Fig. S4A-J). By contrast, the N-terminal region, IKKε-interacting region and Ctp-interacting region of Spn-F were dispensable for its tip localization (Fig. 5A-E,J; supplementary material Fig. S4A-J). These results suggested that Spn-F is transported to the tip of growing bristles by cytoplasmic dynein, independent of its ability to bind Ctp.

Rescue analyses of the *spn-F^1^* bristle morphology phenotype revealed that Spn-F must be able to bind IKKε and cytoplasmic dynein simultaneously to support bristle morphogenesis. The expression of full-length Spn-F rescued the *spn-F^1^* bristle phenotype (Fig. 5F), whereas the construct lacking the IKKε-binding region (Δ212) failed to rescue (Fig. 5H; supplementary material Fig. S4A′-H′,K). These results suggested that the ability of Spn-F to bind IKKε is essential for Spn-F function in bristle elongation (Fig. 5K). By contrast, a Spn-F mutant that lacked the dynein-binding region (ΔC2) could partially suppress the *spn-F^1^* bristle phenotype (Fig. 5G), which was relatively normal in the proximal region but disorganized at the distal tip (Fig. 5G′). On the other hand, a construct lacking the Ctp-binding region (Δ253) rescued the *spn-F^1^* bristle phenotype (Fig. 5I), suggesting that the ability of Spn-F to interact with cytoplasmic dynein via its C-terminus, but not through Ctp, is important in bristle morphogenesis. Ctp nevertheless participates in bristle morphogenesis, probably by supporting dynein function, as both *ctp* and *Dhc64C* mutant bristles are reported to be short and thin (Phillis et al., 1996; Dick et al., 1996; Gepner et al., 1996).

Taken together, these results demonstrated that the ability of Spn-F to simultaneously bind IKKε and cytoplasmic dynein is essential for bristle morphogenesis.

### Jvl maintains Spn-F–IKKε at the distal tip of growing bristles

**Fig. 6.**
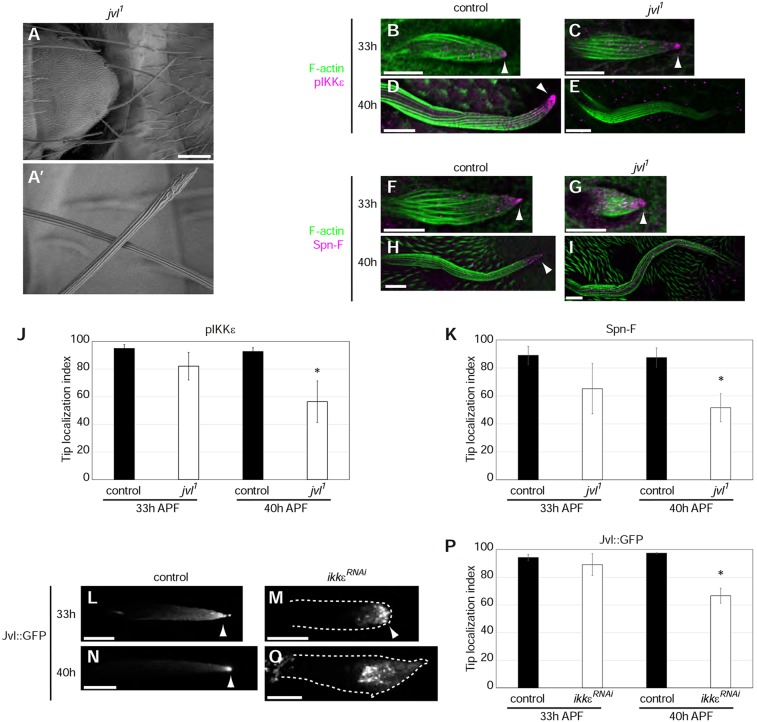
**Jvl is required to maintain IKKε and Spn-F at the distal tip.** (A) SEM image of scutellar bristles in *jvl^1^* mutants. (A′) Magnified image of the *jvl^1^* bristle morphology. (B-I) Localization of pIKKε (magenta, B-E) and Spn-F (magenta, F-I) in control and *jvl^1^* bristles. Bristle morphology is shown by F-actin (green). pIKKε and Spn-F localize to the tip (arrowheads) of control and *jvl^1^* bristles at 33 h APF. At 40 h APF, pIKKε and Spn-F localize to the tip (arrowheads) in control bristles, but are greatly reduced in *jvl^1^* bristles. (J,K) Quantification of the tip localization of pIKKε (J) and Spn-F (K). Controls at 33 h APF are identical to those in Fig. 2I,J. (L-O) Localization of Jvl::GFP in control and *ikkε^RNAi^* bristles. Dotted lines (M,O) outline the cell. (P) Quantification of Jvl::GFP tip localization. Error bars indicate s.d.; *n*=3-4 bristles analyzed. *P<0.05. Scale bars: 100 µm in A; 10 µm in B-I,L-O.

The above results demonstrated that Spn-F acts as a cargo adaptor to link IKKε to cytoplasmic dynein. Since Spn-F is stably localized to the distal tip, we expected that the Spn-F–IKKε complex would be selectively retained at the distal tip. To clarify the molecular mechanisms of Spn-F retention we focused on Jvl, which is reported to interact with Spn-F and has been implicated in IKKε polarization in developing oocytes (Dubin-Bar et al., 2011; Amsalem et al., 2013). *jvl^1^* mutant bristles show a disorganized distal tip morphology (Dubin-Bar et al., 2011) (Fig. 6A), similar to the bristle defect observed in *spn-F^1^* mutants rescued by the dynein-binding-deficient mutant (ΔC2, Fig. 5G).

In *jvl^1^* bristles, pIKKε and Spn-F localized to the distal tip at the early stage of elongation (33 h APF), indicating that Jvl is dispensable for initially targeting the Spn-F–IKKε complex to the distal tip (Fig. 6B,C,F,G). However, at later stages of elongation (40 h APF), pIKKε and Spn-F were no longer concentrated at the distal tip, demonstrating that Jvl is essential for maintaining the Spn-F–IKKε complex at the distal tip (Fig. 6D,E,H-K). Conversely, Jvl::GFP was localized to the distal tip in *ikkε^RNAi^* bristles at the early stage of elongation (33 h APF), indicating that Jvl and the Spn-F–IKKε complex are transported independently to the distal tip (Fig. 6L,M). At later stages of elongation (40 h APF), Jvl::GFP lost its tip localization in the *ikkε^RNAi^* bristles, consistent with IKKε roles in cell polarity maintenance (Fig. 6N-P). These results suggested that Jvl is required to retain Spn-F–IKKε at the distal tip during bristle cell elongation.

### Jvl and Spn-F immobilize each other in S2 cells

**Fig. 7.**
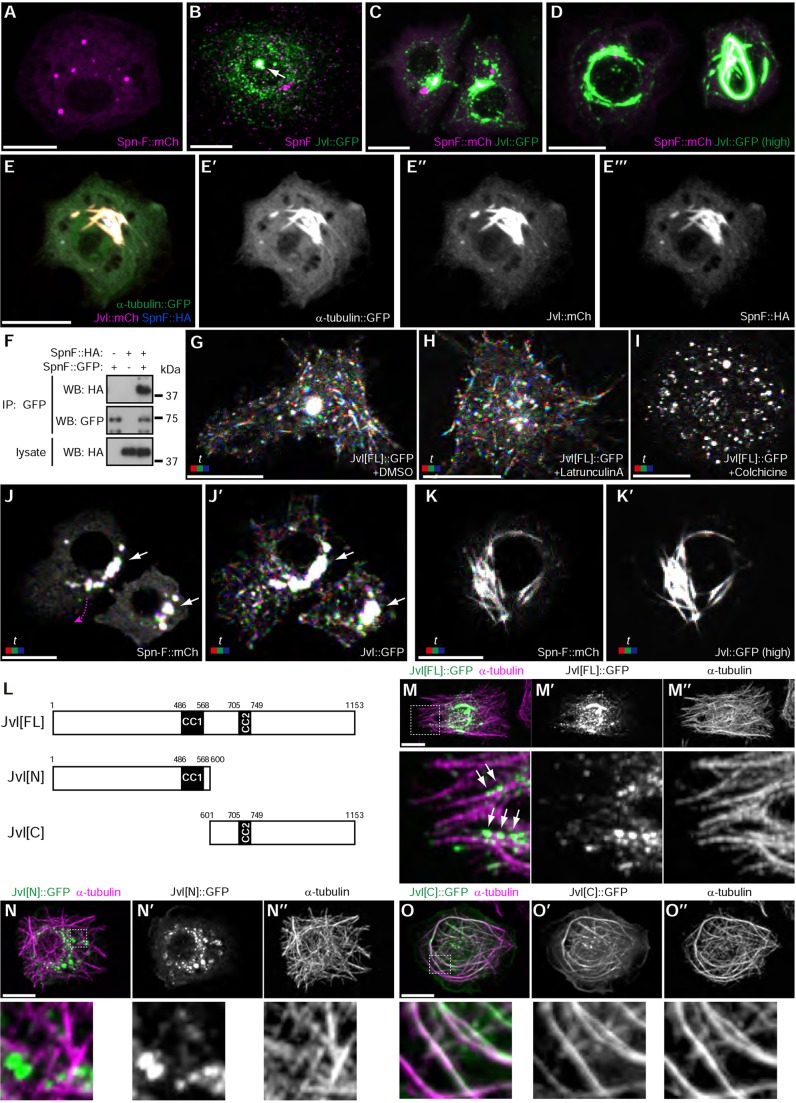
**Jvl and Spn-F immobilize each other in S2 cells.** (A,B) Localization of (A) SpnF::mCh (magenta) and (B) Jvl::GFP (green) to punctate cytoplasmic structures in S2 cells. The large puncta of Jvl::GFP (arrow) colocalize with endogenous Spn-F (magenta). (C) SpnF::mCh (magenta) and Jvl::GFP (green) partially colocalize. (D) Overexpressed SpnF::mCh (magenta) and Jvl::GFP (green) colocalize and form filamentous structures. (E) α-tubulin-GFP (green) colocalizes with Jvl::mCh (magenta) and Spn-F::HA (blue). (F) Spn-F forms oligomers in S2 cells. (G-I) Mobility of Jvl::GFP depends on microtubules. S2 cells expressing Jvl::GFP were treated with DMSO (G), 1 µM Latrunculin A (H) or 10 µM Colchicine (I). (J) At moderate expression levels, SpnF::mCh/Jvl::GFP double-positive particles (white arrows) are immobile, whereas Jvl::GFP or SpnF::mCh (magenta arrow) single-positive particles can move. (K) Upon overexpression, Jvl::GFP/SpnF::mCh double-positive structures are immobile. Red, 0 s; green, 3 s; blue, 6 s. (L) The Jvl constructs used in M-O. (M-O) Microtubule (magenta) localization of Jvl deletion mutants (green) in S2 cells. Jvl[FL]::GFP puncta localized along microtubules (M, arrows), whereas Jvl[N]::GFP puncta did not overlap with microtubules (N). Jvl[C]::GFP uniformly decorated microtubules (O). See also supplementary material Movies 9-13. Scale bars: 10 µm.

To clarify the relationship between Spn-F and Jvl, we expressed them in S2 cells. Spn-F::mCh and Jvl::GFP localized to punctate structures in S2 cells (Fig. 7A,B), and Jvl::GFP formed relatively large puncta at the center of the cell where it colocalized with endogenous Spn-F (Fig. 7B). Coexpression of the two proteins at low levels resulted in their partial colocalization at cytoplasmic punctate structures (Fig. 7C), whereas co-overexpression resulted in the formation of filamentous bundles, where Spn-F::mCh and Jvl::GFP colocalized (Fig. 7D). These structures colocalized with α-tubulin::GFP, suggesting that they were microtubule bundles, consistent with previous observations (Dubin-Bar et al., 2011) (Fig. 7E). The ability of Spn-F to form oligomers (Fig. 7F) suggests that Spn-F and Jvl could form higher-order complexes, although we cannot completely rule out the possibility that the large Spn-F/Jvl-containing puncta observed in S2 cells are aggregates formed by overexpression.

Time-lapse imaging revealed that the Jvl::GFP puncta were highly dynamic (Fig. 7G; supplementary material Movie 9). This mobility was abolished by Colchicine (Fig. 7I; supplementary material Movie 11) but not by Latrunculin A (Fig. 7H; supplementary material Movie 10), suggesting that Jvl::GFP mobility depended on microtubules. In contrast to the majority of Jvl::GFP puncta, the large Jvl::GFP-positive puncta that colocalized with endogenous Spn-F (Fig. 7B) were immobile (Fig. 7G). To examine how the interaction of Spn-F and Jvl affected their mobility, we coexpressed Spn-F::mCh and Jvl::GFP and performed time-lapse imaging (supplementary material Movie 12). Intriguingly, Spn-F::mCh/Jvl::GFP double-positive puncta were immobile (Fig. 7J, white arrow), whereas Spn-F::mCh or Jvl::GFP single-positive puncta within the same cell were able to move (Fig. 7J, magenta arrow for Spn-F::mCh). Overexpression of Spn-F::mCh and Jvl::GFP completely immobilized the two molecules (Fig. 7K; supplementary material Movie 13). These results suggested that Spn-F and Jvl immobilize each other in S2 cells.

### Jvl interacts with microtubules through its C-terminal region

To elucidate how its binding to microtubules is regulated, we performed a structure-function analysis of Jvl (Fig. 7L). Full-length Jvl localized to punctate structures that were located along microtubules (Fig. 7M). The N-terminal half of Jvl (Jvl[N]) localized to punctate structures, but failed to colocalize with microtubules (Fig. 7N). By contrast, the C-terminal half of Jvl (Jvl[C]) uniformly decorated microtubules (Fig. 7O). These results suggested that Jvl interacts with microtubules through its C-terminal region.

## DISCUSSION

### The distal tip acts as a sorting station for cytoplasmic dynein-dependent cargoes

**Fig. 8.**
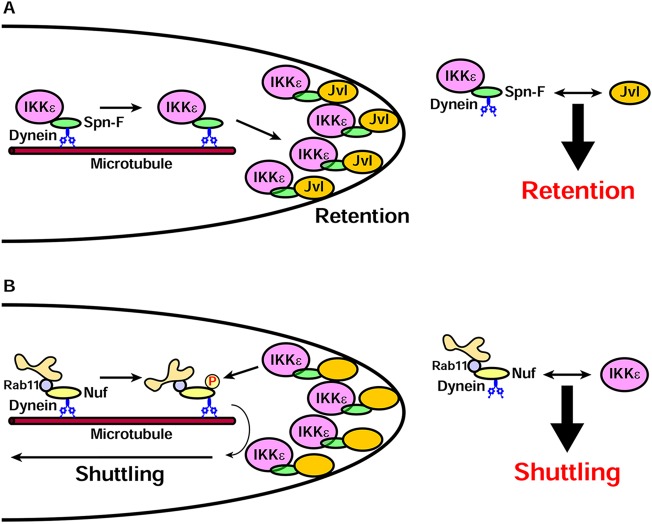
**Model for bristle tip IKKε–Spn-F transport and retention.** (A) Transport and retention of the IKKε–Spn-F complex. The IKKε–Spn-F complex is transported to the distal tip by cytoplasmic dynein. Jvl is independently transported to the distal tip and interacts with Spn-F to retain the IKKε–Spn-F complex there. (B) The shuttling movement of recycling endosomes. Rab11-positive recycling endosomes are transported by cytoplasmic dynein to the distal tip. IKKε phosphorylates Nuf, a Rab11-dynein adaptor protein, and promotes motor switching. Specific pairings between cargo adaptors (Spn-F, Nuf) and regulatory molecules (Jvl, IKKε) determine cargo fate.

Here we demonstrated that the bristle tip is a sorting station for cytoplasmic dynein-dependent cargoes. The IKKε–Spn-F complex, which acts as the signaling center in bristle cell elongation, localizes to the distal tip by dynein-dependent polarized transport and Jvl-dependent selective retention (Fig. 8A). By contrast, Rab11-positive recycling endosomes undergo both dynein-dependent distal transport and proximal transport, which is probably mediated by kinesins (Fig. 8B) (Gould, 2011; Otani et al., 2011).

The distinct transport characteristics at the distal tip are specified by the nature of the adaptor proteins. IKKε is transported to the distal tip by dynein via the adaptor protein Spn-F, and the IKKε–Spn-F complex is stably retained at the distal tip by Jvl, a Spn-F-interacting protein. By contrast, Rab11-positive recycling endosomes are transported to the distal tip by dynein via the adaptor protein Nuf, where it is phosphorylated by IKKε (Otani et al., 2011). This phosphorylation inactivates the dynein-dependent transport of Rab11-positive recycling endosomes, thereby promoting their transport back to the cell body (Otani et al., 2011). Thus, the IKKε–Spn-F complex stably localizes to the distal tip by polarized transport followed by selective retention, whereas Rab11-positive recycling endosomes bidirectionally shuttle by polarized transport and motor switching. The pivotal step in this sorting decision is the specific recognition of the cargo adaptor proteins (Spn-F and Nuf) by their regulatory proteins (Jvl and IKKε) at the distal tip. These results support the emerging concept that cargo adaptor proteins are not merely physical linkers between cargoes and motors, but act as regulatory hubs where various signals converge (Fu and Holzbaur, 2014).

### Jvl retains the IKKε–Spn-F complex at the distal tip

We identified Jvl as a key regulator of IKKε–Spn-F retention at the distal tip. Jvl interacts with microtubules (Dubin-Bar et al., 2011), and binding Spn-F promotes the microtubule binding activity of Jvl and induces microtubule bundling in S2 cells. Full-length Jvl localizes to punctate structures that were located along microtubules, whereas the C-terminal half of Jvl uniformly decorated microtubules. These results imply that Jvl microtubule binding activity is repressed by its N-terminal region, and that binding Spn-F could relieve this inhibition. Oligomerization of Spn-F could promote the formation of higher-order Spn-F–Jvl complexes to generate multivalent microtubule-binding sites, thereby increasing the microtubule binding activity of Jvl.

Spn-F and Jvl are independently transported to the distal tip in elongating bristles, indicating that their interaction occurs upon arrival at the tip. This interaction presumably activates Jvl microtubule binding activity, which then serves as a molecular brake to immobilize the complex on microtubules. Similar mechanisms have been proposed for the anchoring of mitochondria by Syntaphilin and Kinesin-1 in axonal mitochondrial transport (Chen and Sheng, 2013; Kang et al., 2008), and for the immobilization of lysosomes in dendrites by the interaction of TMEM106B and MAP6 (Schwenk et al., 2014). The coupling of cargo adaptor proteins with microtubule-binding proteins might be a general mechanism for regulating the transport of a particular cargo in a spatiotemporally controlled manner. As Spn-F and Jvl are also involved in the polarized activation of IKKε during oogenesis (Amsalem et al., 2013), similar mechanisms might help generate and maintain cell polarity in various cell types.

As an alternative to the molecular brake model, Jvl could act as a scaffolding protein to recruit enzymes that modify the IKKε–Spn-F complex to promote its retention, or as a regulator of microtubule organization at the distal tip to maintain the polarized organization of the cytoskeleton during bristle elongation. Further analysis of the molecular functions of Jvl will help in elucidating the mechanisms of IKKε–Spn-F retention.

### Spn-F regulates the localization and function of IKKε

Our results suggest that Spn-F functions as a cargo adaptor for IKKε and cytoplasmic dynein. Structure-function analysis of Spn-F demonstrated that its dynein-binding region is required for localizing IKKε to the distal tip and for bristle morphogenesis. In contrast to the dynein-binding-deficient Spn-F mutant, which partially suppressed the *spn-F* mutant bristle morphology phenotype, a mutant lacking the IKKε-binding region completely failed to rescue, indicating that, in addition to its function as a cargo adaptor, Spn-F has a role in regulating IKKε activity. This role could involve regulating IKKε kinase activity or protein stability, or in scaffolding the components of the IKKε signaling pathway. IKKε overexpression could partially suppress the *spn-F* mutant bristle morphology phenotype despite IKKε delocalization from the distal tip, suggesting that increasing the dosage of IKKε can compensate for the loss of Spn-F to some extent. It is likely that the delocalized IKKε can phosphorylate some of its downstream target molecules (such as Nuf and Diap1) to partially support bristle morphogenesis (Kuranaga et al., 2006; Otani et al., 2011).

### Conclusions

In summary, we have demonstrated that the signaling center for bristle elongation is localized to the distal tip by polarized transport and selective retention mechanisms. The distal tip of bristles acts as a sorting center for cytoplasmic dynein cargoes, where regulatory proteins recognize cargo adaptor proteins and determine whether cargo is retained or sent back to the cell body. These findings support the idea that cargo adaptor proteins act as regulatory hubs where various signals converge. It would be interesting to test whether the differential regulation of cargo-motor interactions contributes to the formation of signaling centers during the morphogenesis of mammalian cells of complex shape, such as neurons and podocytes.

## MATERIALS AND METHODS

### Molecular biology

*spn-F* cDNA was cloned by RT-PCR from S2 cells. *ctp* cDNA was from the Drosophila Genomics Resource Center (clone LD24056). *jvl* cDNA (Dubin-Bar et al., 2011), IKKε[WT] and IKKε[K41A] constructs were characterized previously (Oshima et al., 2006). The Spn-F (ΔN, ΔC2, N, M, C, N1, N2, C1, C2) and Jvl (N, C) deletion mutants were generated by PCR; ΔC1 was generated by inserting the corresponding annealed synthetic oligonucleotide into the *Bst*EII/*Bst*XI sites of *spn-F* (Hokkaido System Science); Δ191, Δ212, Δ231, Δ253 were generated by synthesizing the corresponding gene fragments and subcloning them into the *Bst*EII/*Bst*XI sites of *spn-F* (GenScript). Fusion constructs were generated by subcloning *ikkε*, *spn-F*, *ctp* and *jvl* into pUAST-EGFP-N, pUAST-mCh, pUAST-myc-Ntag, pUAST-myc-Ctag, pUAST-HA-Ctag or pGEX-6P1 (GE Healthcare) vectors (Brand and Perrimon, 1993). The Ctp expression vectors were constructed by Ken Kakihara and the pUAST-α-tubulin::GFP vector was generated by Atsushi Wada in our laboratory.

### *Drosophila* stocks

The following *Drosophila* strains were used: *y^1^ w^67C21^* as a control; *spn-F^1^* (Abdu et al., 2006), *ikkε^66^* (Oshima et al., 2006), *ikkε^RNAi^* (Oshima et al., 2006), *ikkε^DN^* (Oshima et al., 2006) and *jvl^1^* (Dubin-Bar et al., 2011) were described previously; *ikkε^alice^* was provided by Kathryn Anderson (Shapiro and Anderson, 2006); *Dhc64C^RNAi^* (P{GD12258}v28054) was from the Vienna Drosophila RNAi Center; and *UAS-HA::Dhc64C* was provided by Tom Hays (Silvanovich et al., 2003). *Drosophila* were raised at 25°C with the following exceptions: 16-20°C for IKKε overexpression by the *Sca-Gal4 tub-Gal80^ts^* driver, and 30-32°C for *Dhc64C* RNAi by the *neu-Gal4 tub-Gal80^ts^*driver.

Transgenic flies were generated by standard P-element-mediated transgenesis, and overexpression was performed using the Gal4-UAS system (Brand and Perrimon, 1993). *Sca-Gal4* (de Celis et al., 1999), *neu-PGal4-72* (a kind gift from François Schweisguth, Institut Pasteur, Paris, France) (Bellaiche et al., 2001), *neu-PGal4-72 tub-Gal80^ts^* (provided by Adrian Moore, RIKEN-BSI, Japan) and *Sca-Gal4 tub-Gal80^ts^* (generated by recombination) were used for overexpression; and *tub-Gal80^ts^* (McGuire et al., 2003) was from the Bloomington Stock Center. *ikkε* mutant clones were generated by the FLP-FRT system (Xu and Rubin, 1993) using *Ubx-flp* (a kind gift of Jürgen Knoblich) (Emery et al., 2005), and transgenes were expressed in mutant clones by the mosaic analysis with a repressible cell marker (MARCM) system (Lee and Luo, 1999). See supplementary material Table S1 for the genotypes used in each experiment.

### Antibodies

Guinea pig and rabbit anti-Spn-F N-terminus antibodies were generated by injecting purified GST-Spn-F-N (amino acids 1-190) into guinea pigs and rabbits. The immunization and affinity purification by antigen-conjugated column were performed by MBL. The mouse anti-Spn-F antibody (8C10) (Abdu et al., 2006), mouse anti-IKKε antibody (clone #80) (Oshima et al., 2006) and affinity-purified rabbit anti-pIKKε antibody (S175) (Otani et al., 2011) were described previously. Mouse anti-dynein heavy chain monoclonal antibody (clone 2C11-2) was from the Developmental Studies Hybridoma Bank (Sharp et al., 2000). See supplementary material Table S2 for a full description of the antibodies used.

### Cell culture and immunofluorescence

*Drosophila* S2 cells were cultured in Schneider's Insect Medium (Gibco) supplemented with 10% FCS and antibiotics at 25°C (Schneider, 1972). pUAST vectors with actin5Ce-Gal4 drivers were cotransfected using Effectene (Qiagen) according to the manufacturer's instructions, and harvested 36-48 h after transfection. For immunofluorescence or time-lapse imaging, cells were replated on coverslips or glass-bottom dishes coated with Concanavalin A (Wako) and were allowed to spread for 1-2 h (Rogers et al., 2002). For drug treatments, cells were treated with 1 µM Latrunculin A (Wako) or 10 µM Colchicine (Wako) for 1 h before imaging. For immunofluorescence, cells were fixed in 4% paraformaldehyde in phosphate-buffered saline (PBS) for 20 min at room temperature, permeabilized with 0.1% Triton X-100 in PBS (PBS-T) for 15 min, and blocked with 5% skimmed milk in Tris-buffered saline (TBS). Primary and secondary antibodies were diluted in the blocking solution. After each antibody incubation, the coverslips were washed three times with PBS-T. The cells were mounted in Vectashield mounting medium (Vector Labs).

### Immunohistochemistry

Pupae were fixed as described previously (Otani et al., 2011). Blocking was performed in 0.1% BSA, 0.2% Triton X-100 and 0.2% Tween 20 in PBS overnight at 4°C. Primary and secondary antibodies were diluted in the blocking solution and incubated with the sample overnight with gentle agitation at 4°C. Samples were washed with PBS-T three times after antibody incubation steps. The thorax pieces were mounted dorsal side up on glass slides in Vectashield mounting medium and covered with a coverslip; a second coverslip was used as a spacer.

### Confocal microscopy

Confocal microscopy was performed on an FV1000-BX61 laser-scanning confocal microscope using an UPlanSApo 60×/NA 1.35 objective (all Olympus). Movies were captured using the FV1000-IX81 microscope using a PlanApo N 60×/NA 1.42 objective (all Olympus). Macrochaetes were imaged for all experiments. *z*-stack image generation and brightness and contrast adjustment were performed using ImageJ (NIH) without any nonlinear adjustments. Gaussian filter was applied to generate still images from time-lapse imaging of S2 cells.

The ‘tip index’ was determined as follows (see also supplementary material Fig. S1). A line scan was performed from the base of the bristle to the distal tip to obtain a plot profile using ImageJ. Subsequent analyses were performed using Excel (Microsoft). The maximum intensity (100% intensity) and bristle length (Position[Max]) were determined from the line scan, and pixels that exceeded 50% intensity were identified. The tip index was defined as the relative position of the pixels that exceeded 50% intensity along the proximal-distal axis of the bristle; the full bristle length was defined as 100. Statistical analyses (Student's *t*-test) were performed using Excel.

### Scanning electron microscopy (SEM)

Adult flies were anesthetized by CO_2_ and the legs and wings were removed by fine forceps. The dissected flies were mounted dorsal side up, sputter-coated with platinum (JFC-1600; JEOL) or osmium (Neoc-STB; Meiwafosis) and viewed with a scanning electron microscope (JSM-5600-LV; JEOL) at low vacuum (30 Pa) using an acceleration voltage of 10 kV. Scutellar bristles were imaged for all experiments. The scutellar bristle length was measured by ImageJ, and statistical analyses (Student's *t*-test) were performed using Excel.

### Biochemistry

Transfected S2 cells were lysed in lysis buffer (50 mM Tris-HCl pH 7.5, 150 mM NaCl, 0.5% Triton X-100, 10% glycerol, 1 mM EDTA, 1 mM DTT). To generate ovary extracts, adult female flies were cultured on yeast for 3 days. The flies were anesthetized by CO_2_ and the ovaries were dissected under PBS. The dissected ovaries from 40 control females or 100 *spn-F^1^* mutant females were then homogenized in 1 ml lysis buffer; more *spn-F^1^* mutant ovaries were used because they were underdeveloped due to oogenesis defects (Abdu et al., 2006). The lysates were incubated for 30 min at 4°C, then cleared by centrifugation at 20,000 ***g*** for 10 min at 4°C. Anti-HA beads (clone 3F10, Roche), anti-GFP beads (MBL) or anti-myc beads (clone PL14, MBL) were then added to the supernatant and the samples were incubated with rotation for 2 h at 4°C. Alternatively, rabbit anti-Spn-F or rabbit anti-GFP (as control IgG) antibodies were added to the supernatant, and after 1 h incubation with rotation at 4°C the samples were further incubated for 1 h with Protein G-Sepharose 4FF beads (GE Healthcare) with rotation at 4°C.

For GST pull-down assays, GST, GST-N, GST-C, GST-C1 and GST-C2 were expressed in BL21 (DE3) pLysS *E. coli* cells (Novagen). Protein expression was induced by adding 0.1 mM IPTG to the bacterial cultures, and proteins were expressed at 20°C for 16-20 h for GST-N, or at 37°C for 3 h for GST, GST-C, GST-C1 and GST-C2. Recombinant protein purification was as described previously (Otani et al., 2011). For the pull-down assays, 20 µg GST-fusion protein was added to ovary lysate, and the mixture was incubated overnight with rotation at 4°C. Glutathione-Sepharose 4B beads (GE Healthcare) were then added, and the samples were incubated with rotation for 2 h at 4°C. The beads were rapidly washed three times with lysis buffer, and the complexes were eluted by boiling in 2× Laemmli sample buffer supplemented with 10% β-mercaptoethanol. To detect interactions with DHC, it is essential that the washes are performed rapidly.

SDS-PAGE was performed by standard methods using 15% (to detect Ctp-myc) or 5-20% SuperSep Ace polyacrylamide gels (Wako). Western blotting was performed as described previously (Otani et al., 2011).

## Supplementary Material

Supplementary Material
