# Supplementary material for: A transport and retention mechanism for the sustained distal localization of Spn-F–IKKε during Drosophila bristle elongation
Source: Development. 2015 Jul 1;142(13):2338–51. doi: 10.1242/dev.121863 (PMC4510591; doi:10.1242/dev.121863)
Supplement: Supplementary Material [file supp_142_13_2338__index.html]

Supplementary Material 

# A transport and retention mechanism for the sustained distal localization of Spn-F–IKKε during *Drosophila* bristle elongation

## DEV121863 Supplementary Material

- Supplementary Material
