# Supplementary material for: A transport and retention mechanism for the sustained distal localization of Spn-F–IKKε during Drosophila bristle elongation
Source: Development. 2015 Jul 1;142(13):2338–51. doi: 10.1242/dev.121863 (PMC4510591; doi:10.1242/dev.121863)
Supplement: Supplementary Material [file supp_dev.121863_DEV121863supp.pdf]

## Supplemental Figures

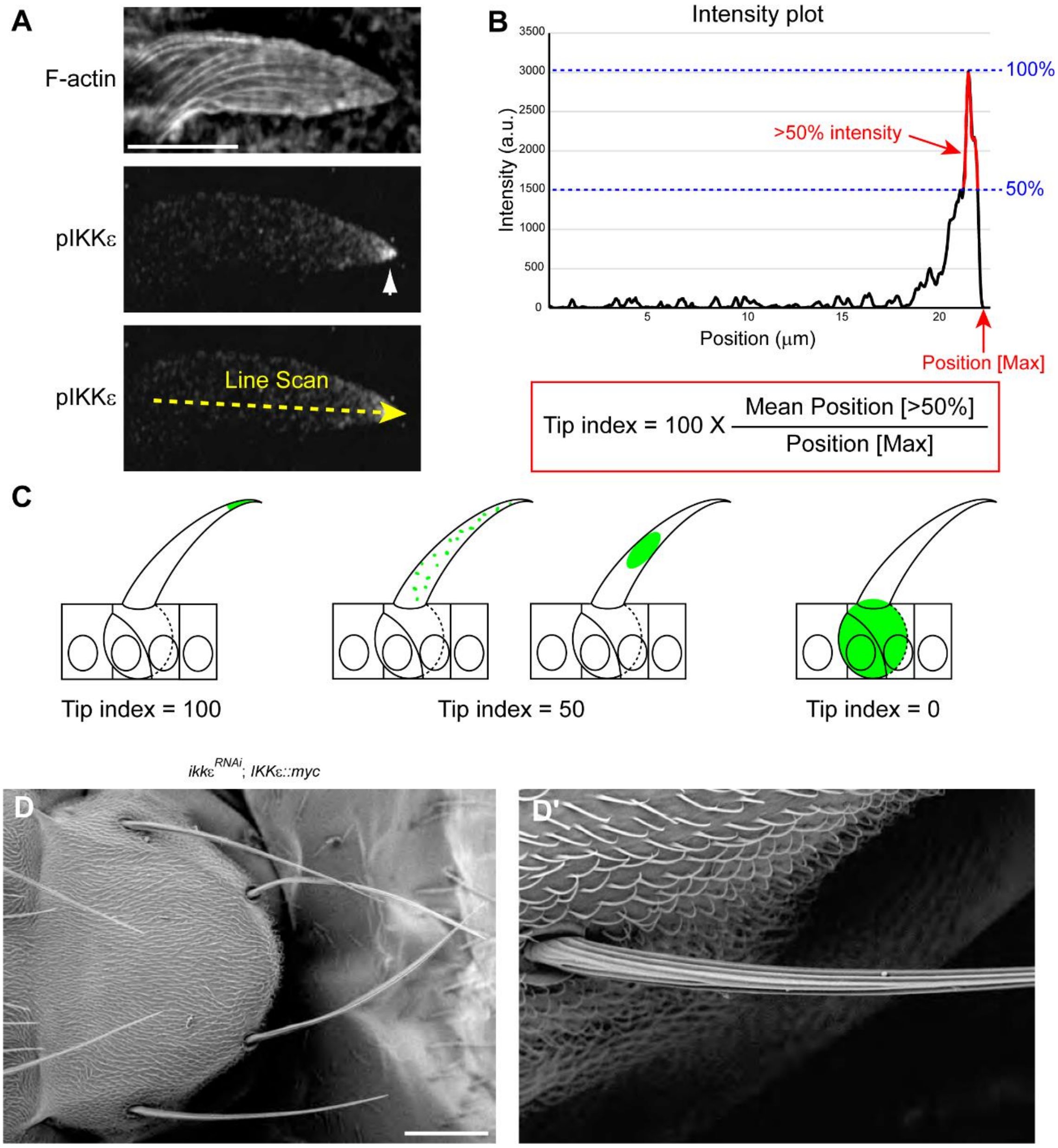


**Figure S1. Tip index measurement and morphology of *ikkε^RNAi*, *IKKε::myc* bristles.**

(A-C) Measurement of the Tip index. (A) F-actin staining by phalloidin was used to determine the outline of the cells. (A') pIKKε accumulated at the distal tip. (A'') A line

scan was performed along the dotted yellow arrow. This picture is identical to Fig. 2D. (B) Intensity plot of the pIKKε staining shown in A. The maximum intensity (100% intensity) and length of the bristles (Position[Max]) were determined from the line scan, and pixels that exceeded 50% intensity (shown in red) were identified. The tip localization index (Tip index) was defined as the relative position of the pixels that exceed 50% intensity along the proximal-distal axis of the bristles, and had a value of 0-100. (C) Examples of Tip indices. If the signals were completely concentrated at the distal tip, the tip index was 100. If the signals were diffuse or accumulated in the middle of the bristle, the tip index was 50. If the signals were completely concentrated at the cell body, the tip index was 0. (D) Coexpression of IKKε::myc and IKKε hairpin RNA resulted in normal bristle morphology. Scale bars, (A) 10 μm, (D) 100 μm.

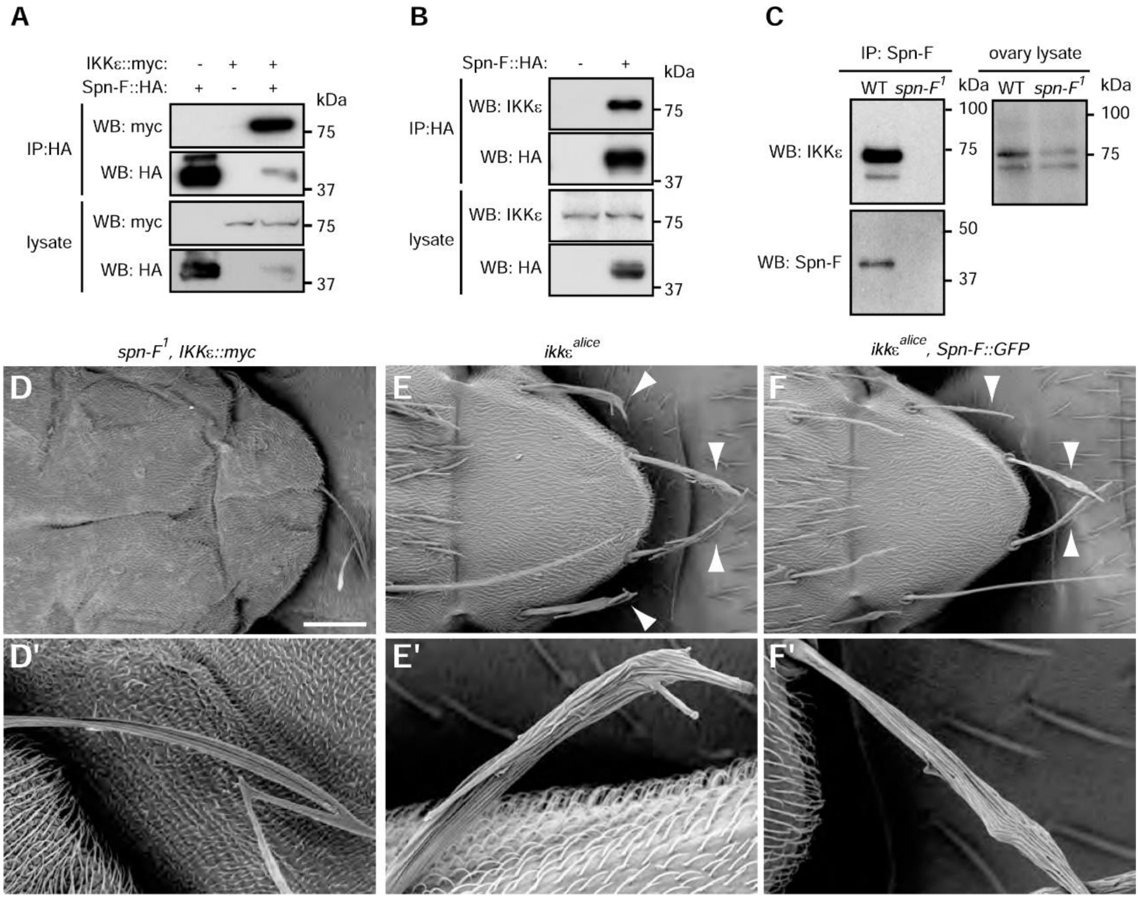


**Figure S2. IKKε interacts with Spn-F.**

(A) Coimmunoprecipitation of IKKε::myc and Spn-F::HA in S2 cells. (B) Coimmunoprecipitation of Spn-F::HA and endogenous IKKε in S2 cells. (C) Coimmunoprecipitation of IKKε with Spn-F in control ovary lysate, but not in *spn-F*$^{1}$ mutant ovary lysate. (D-F) SEM images of scutellar bristles of the indicated genotypes. (D) SEM image of the hooked morphology of IKKε::myc-overexpressing *spnF*$^{1}$ mutant bristles. (E) *ikkε*$^{alice}$ mutant bristles (arrowheads) were short and branched. (F) Spn-F::GFP overexpression in *ikkε*$^{alice}$ mutant bristles (arrowheads) did not suppress the bristle morphology defects. Scale bar, 100 μm.

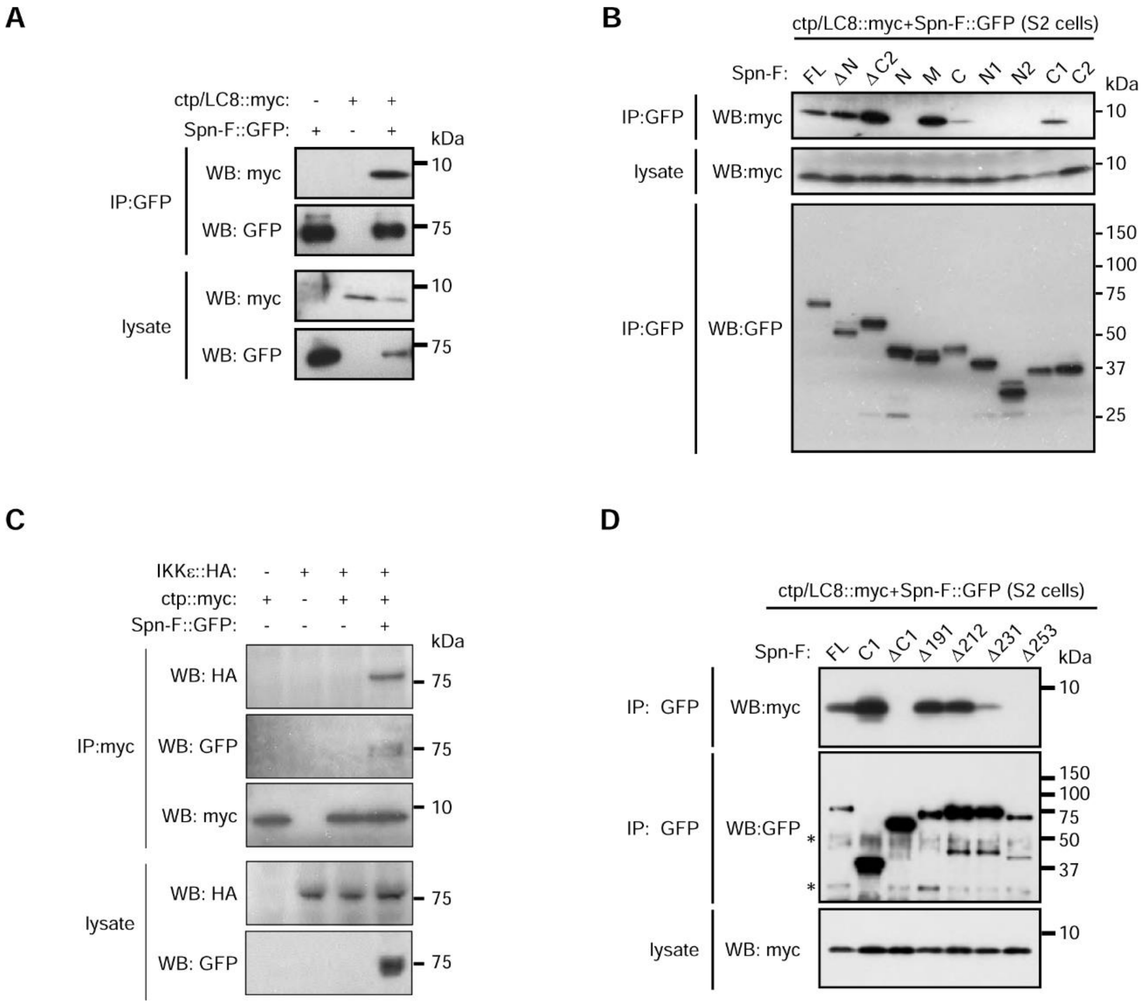


**Figure S3. Ctp/LC8 and IKKε interact with distinct but overlapping regions of Spn-F.**

(A) Coimmunoprecipitation of Ctp/LC8::myc and Spn-F::GFP in S2 cells. (B) Coimmunoprecipitation analysis showing that Spn-F's C1 region was necessary and sufficient to interact with Ctp/LC8::myc. (C) IKKε, Spn-F, and Ctp/LC8 could form a ternary complex in S2 cells. (D) Coimmunoprecipitation analysis showing that amino acids 231-274 of Spn-F were required for its efficient interaction with Ctp/LC8::myc. Asterisks: Immunoglobulin.

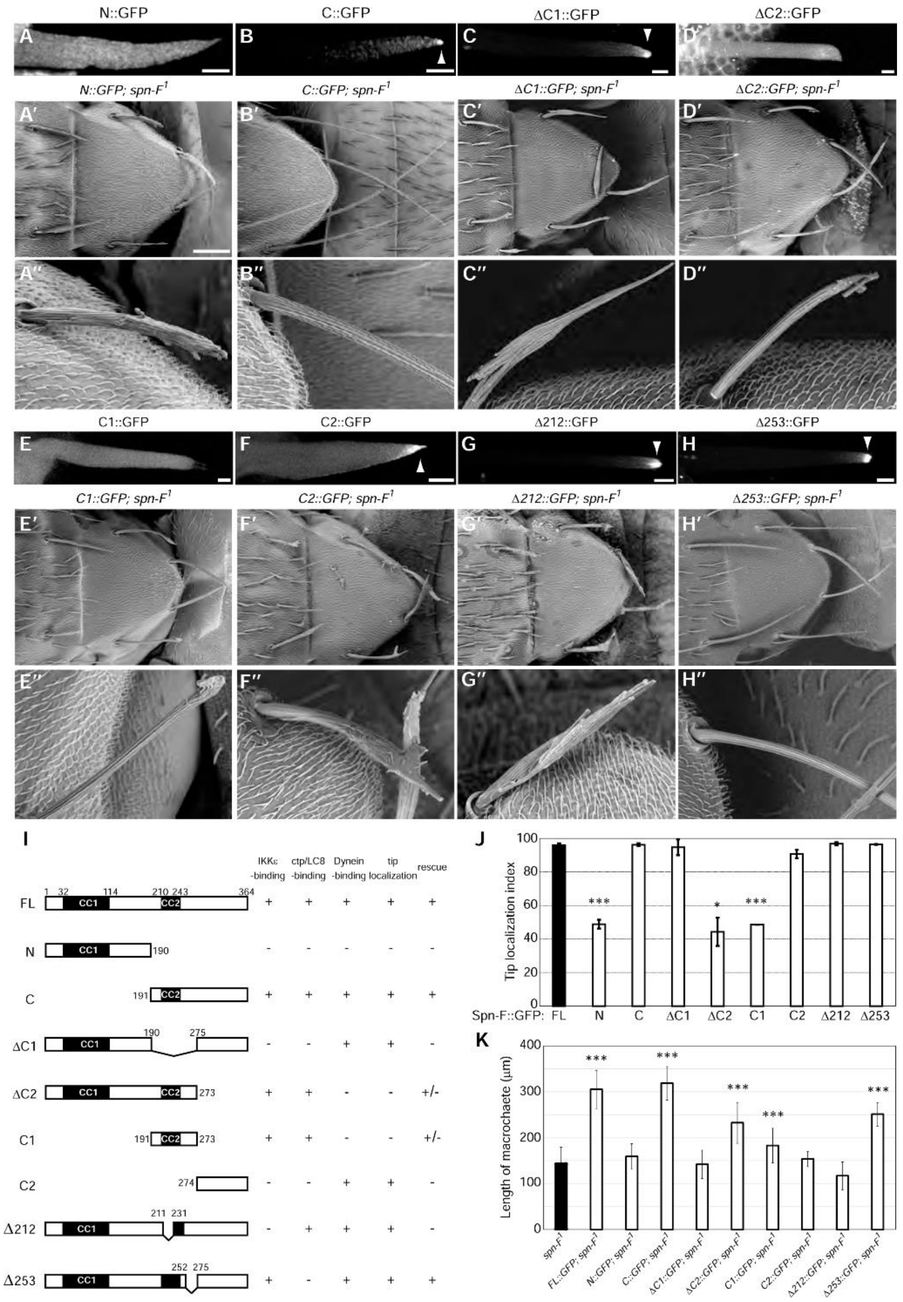


**Figure S4. Spn-F's dynein-interacting region and IKKε-interacting region are both required for bristle morphogenesis.**

(A-H) Localization of Spn-F deletion mutants in developing bristles at 36 h APF.

C::GFP (B), ΔC1::GFP (C), C2::GFP (F), Δ212::GFP (G), and Δ253::GFP (H) localized

to the tip of growing bristles (arrowheads). N::GFP (A), ΔC2::GFP (D), and C1::GFP (E) failed to localize to the tip of growing bristles. (A'-H') SEM images of scutellar bristles of the indicated genotypes. (A''-H'') Magnified images of bristle morphology. C::GFP (B') and Δ253::GFP (H') rescued the *spn-F*$^{1}$ bristle morphology phenotype. N::GFP (A'), ΔC1::GFP (C'), C2::GFP (F'), and Δ212::GFP (G') failed to rescue the *spn-F*$^{1}$ bristle morphology phenotype. ΔC2::GFP (D'') and C1::GFP (E'') partially suppressed the *spn-F*$^{1}$ bristle morphology phenotype. (I) Summary of Spn-F deletion mutants and their ability to rescue the *spn-F*$^{1}$ bristle morphology phenotype. (J) Quantification of the tip localization of Spn-F deletion mutants. *n*=2-4 (bristles analyzed). (K) Quantification of the bristle cell morphology. The length of the macrochaete was measured. Error bars indicate s.d.; *n*>5 (bristles analyzed). *p<0.05, ***p<0.0005. Scale bars, (A-H) 5 μm, (A'-H') 100 μm.

**Supplemental Videos**

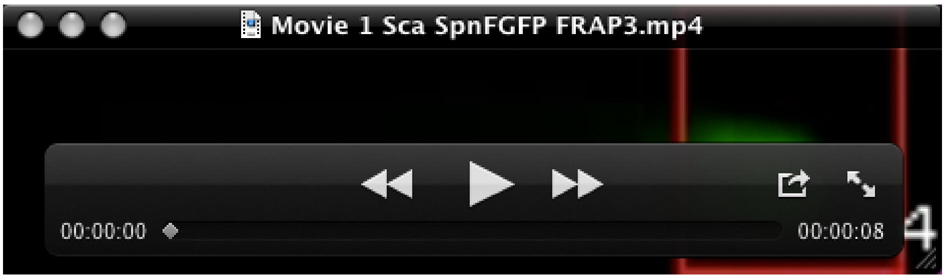


**Movie S1. FRAP analysis of Spn-F::GFP in bristles.**

FRAP analysis of Spn-F::GFP expressing bristles at 33 h APF. Red box indicates the photobleached region. Images were taken at 1 sec interval for 5 min, and the movie shows a single optical section.

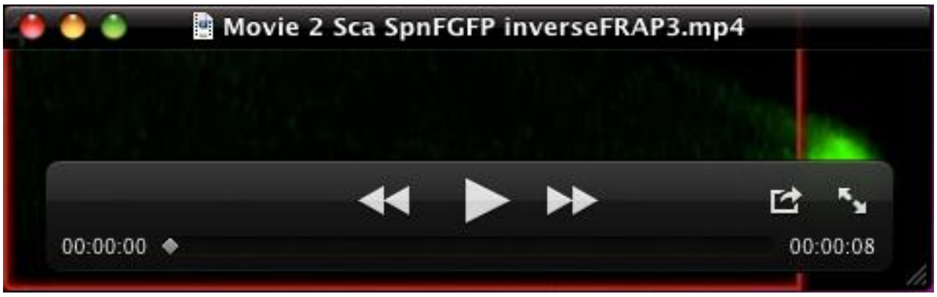


**Movie S2. Inverse-FRAP analysis of Spn-F::GFP in bristles.**

Inverse-FRAP analysis of Spn-F::GFP expressing bristles at 33 h APF. Red box indicates the photobleached region. Images were taken at 1 sec interval for 5 min, and the movie shows a single optical section.

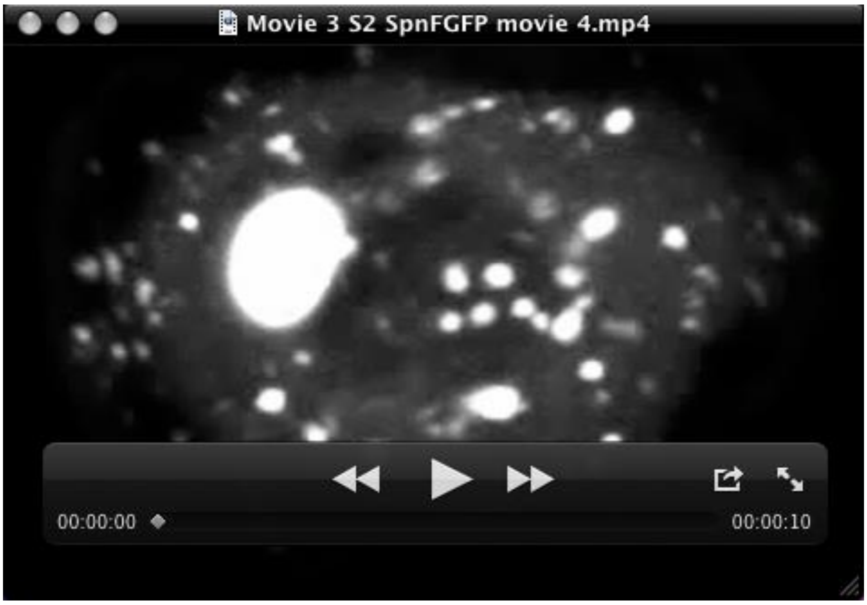


**Movie S3. Mobility of Spn-F::GFP in S2 cells.**

Time-lapse imaging of Spn-F::GFP-expressing S2 cells. Spn-F::GFP localizes to punctate structures that dynamically move within the cytoplasm. Images were taken at 1 sec interval for 5 min, and the movie shows a single optical section.

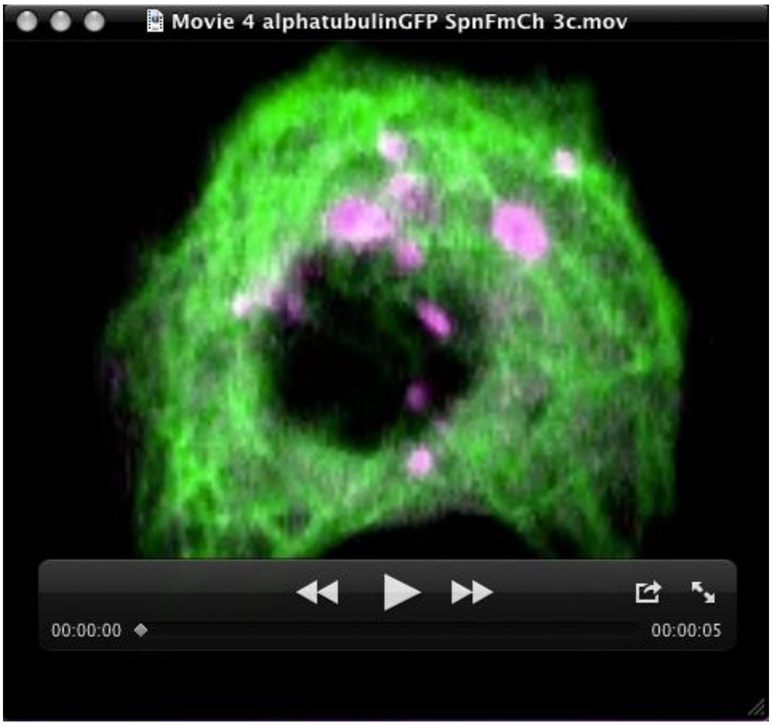


**Movie S4. Spn-F::mCh moves along microtubules.**

Time-lapse imaging of S2 cells expressing α-tubulin::GFP and Spn-F::mCh.

Spn-F::mCh puncta move along the microtubules labeled with α-tubulin::GFP. Images were taken at 1.5 sec interval for 3 min, and the movie shows a single optical section.

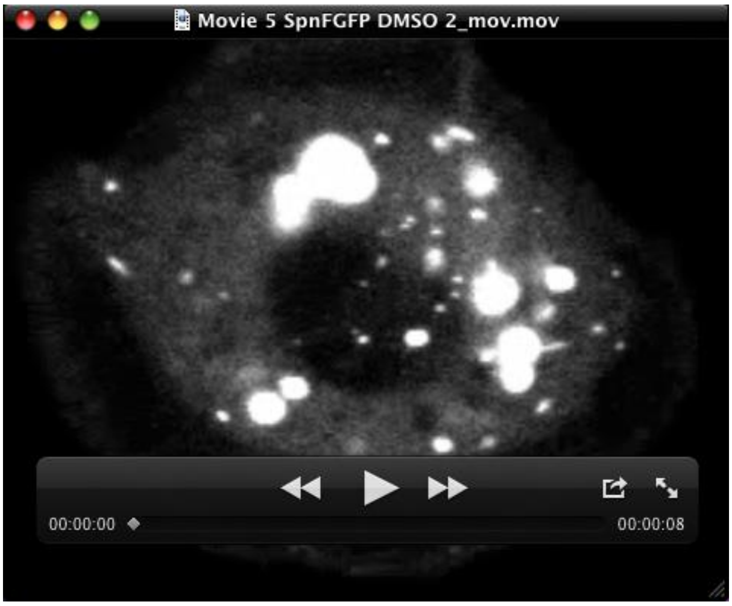


**Movie S5. Spn-F::GFP mobility in DMSO-treated S2 cells**

Time-lapse imaging of Spn-F::GFP-expressing S2 cells treated with DMSO.

Spn-F::GFP puncta dynamically move within the cytoplasm. Images were taken at 1 sec interval for 5 min, and the movie shows a single optical section.

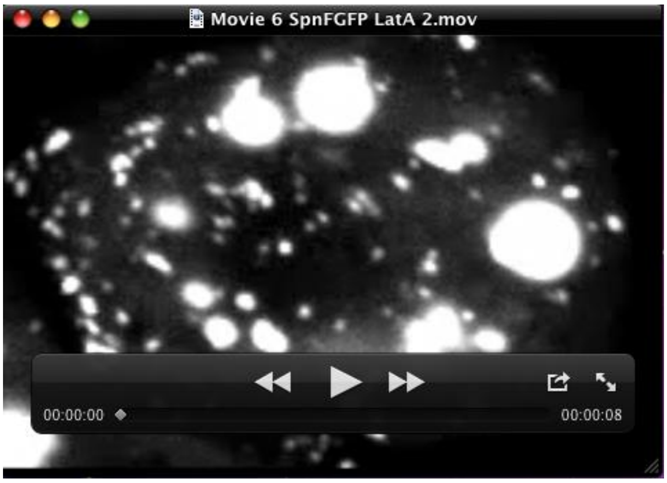


**Movie S6. Spn-F::GFP mobility in LatrunculinA-treated S2 cells.**

Time-lapse imaging of Spn-F::GFP-expressing S2 cells treated with 1 μM LatrunculinA. Spn-F::GFP puncta dynamically move within the cytoplasm. Images were taken at 1 sec interval for 5 min, and the movie shows a single optical section.

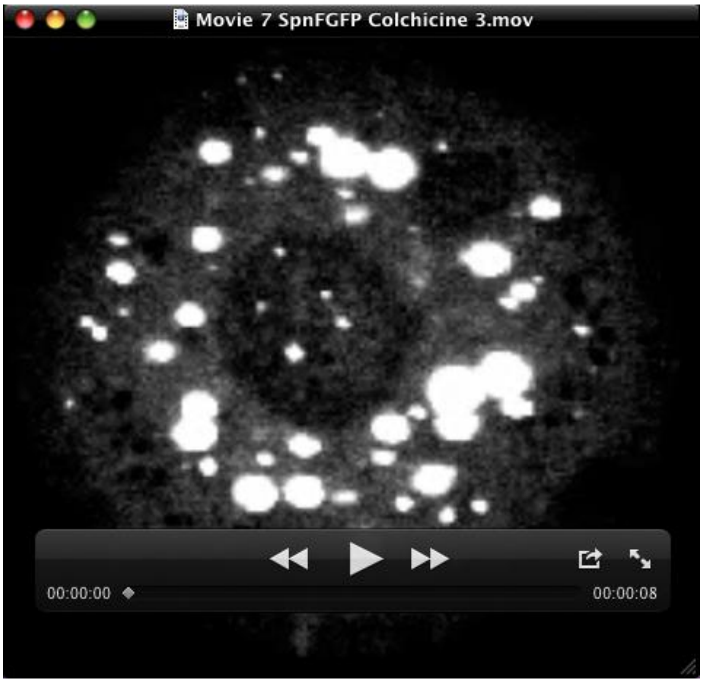


**Movie S7. Spn-F::GFP mobility in colchicine-treated S2 cells.**

Time-lapse imaging of Spn-F::GFP-expressing S2 cells treated with 10 μM colchicine. Spn-F::GFP puncta dynamically move within the cytoplasm. Images were taken at 1 sec interval for 5 min, and the movie shows a single optical section.

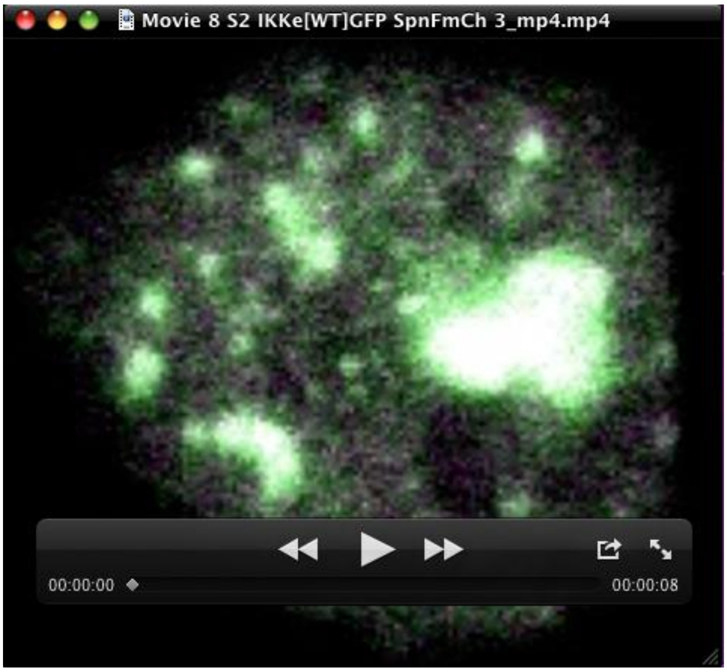


**Movie S8. IKKε::GFP and Spn-F::mCh colocalize and move together.**

Time-lapse imaging of S2 cells expressing IKKε::GFP and Spn-F::mCh. IKKε::GFP and Spn-F::mCh colocalize and move together. Images were taken at 1 sec interval for 3 min, and the movie shows a single optical section.

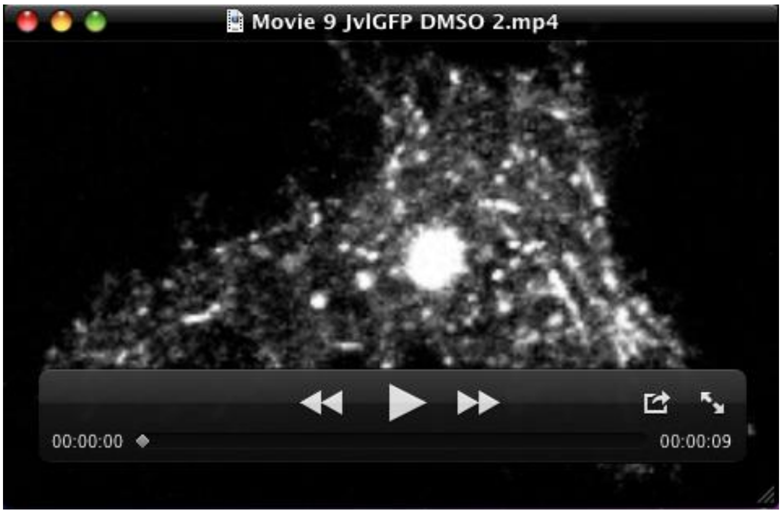


**Movie S9. Jvl::GFP mobility in DMSO-treated S2 cells.**

Time-lapse imaging of Jvl::GFP-expressing S2 cells treated with DMSO. Jvl::GFP puncta dynamically move within the cytoplasm. Images were taken at 1 sec interval for 5 min, and the movie shows a single optical section.

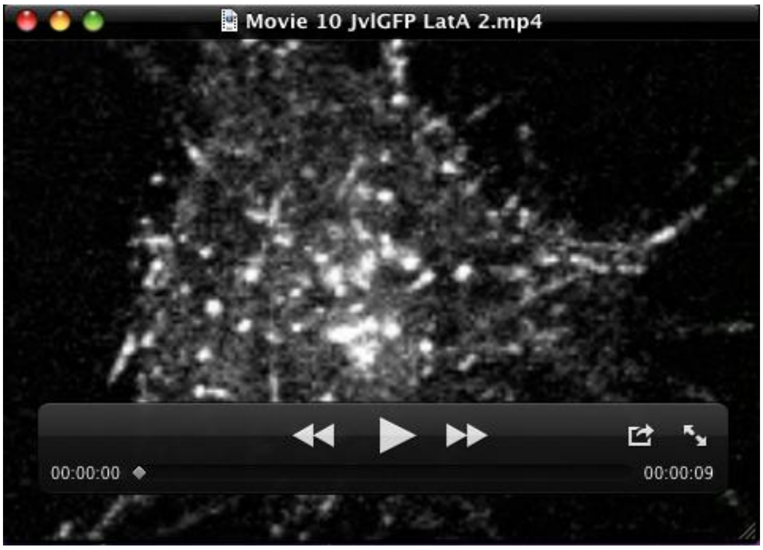


**Movie S10. Jvl::GFP mobility in LatrunculinA-treated S2 cells.**

Time-lapse imaging of Jvl::GFP-expressing S2 cells treated with 1 μM LatrunculinA. Jvl::GFP puncta dynamically move within the cytoplasm. Images were taken at 1 sec interval for 5 min, and the movie shows a single optical section.

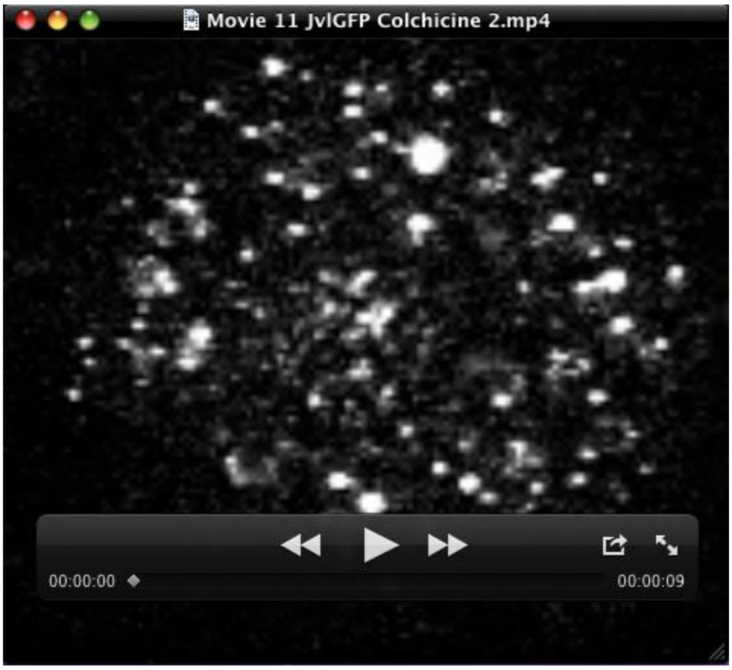


**Movie S11. Jvl::GFP mobility in colchicine-treated S2 cells.**

Time-lapse imaging of Jvl::GFP-expressing S2 cells treated with 10 μM colchicine. Jvl::GFP puncta mobility is suppressed. Images were taken at 1 sec interval for 5 min, and the movie shows a single optical section.

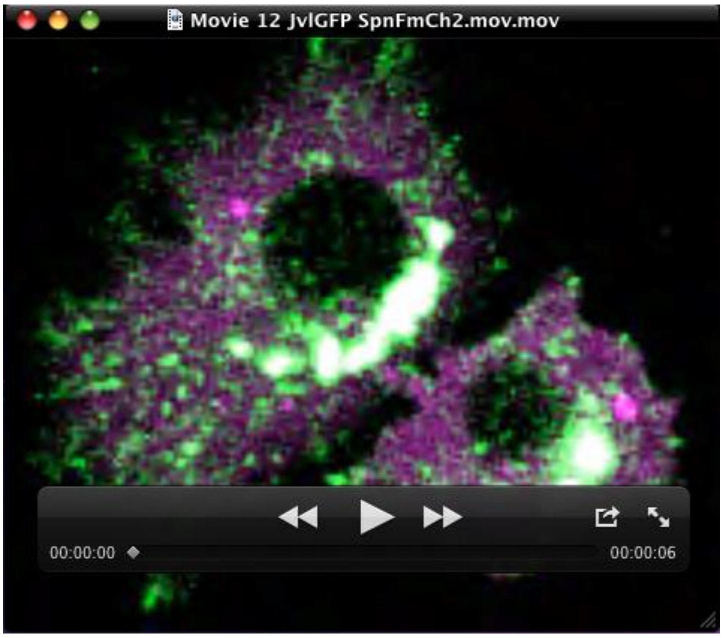


**Movie S12. Spn-F::mCh and Jvl::GFP immobilize each other in S2 cells.**

Time-lapse imaging of S2 cells expressing Jvl::GFP and Spn-F::mCh.

Jvl::GFP/Spn-F::mCh double-positive particles are immobile, whereas Jvl::GFP- or Spn-F::mCh-single-positive particles can move. Images were taken at 1.5 sec interval for 3 min, and the movie shows a single optical section.

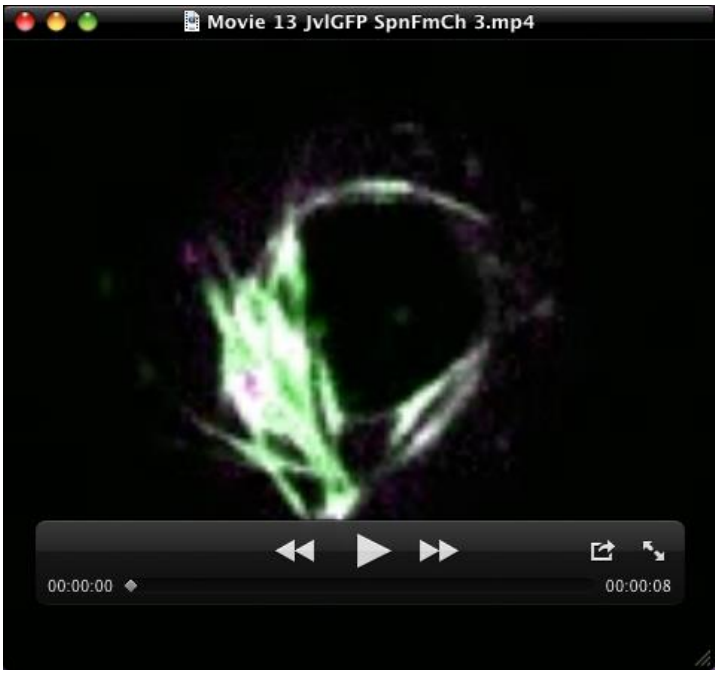


**Movie S13. Spn-F::mCh and Jvl::GFP mobility upon their high-level coexpression in S2 cells.**

Time-lapse imaging of S2 cells expressing Jvl::GFP and Spn-F::mCh at high level. Jvl::GFP and Spn-F::mCh bundle microtubules and are immobile. Images were taken at 1 sec interval for 3 min, and the movie shows a single optical section.

**Table S1. Genotypes of the flies used in this study**

| genotype | Figure numbers |
|---|---|
| *y*$^{1}$ *w*$^{67C21}$ | 1A, 2A, 2D, 2N, 3A, 3B, 4E, 4F, 4H, 6B, 6D, 6F, 6H, S1A, S2C |
| *w; Sca-Gal4/+; UAS-Spn-F::GFP/+* | 1B, 1C |
| *w; Sca-Gal4 IR2615R/CyO hb-lacZ* | 2B, 2E, 3D |
| *w;; spnF*$^{1}$*/spnF*$^{1}$ | 2C, 2F, 3C, 4F, S2C |
| *w; Sca-Gal4 IR2615R/+; UAS-IKKε::myc/+* | 2G, S1D |
| *w; Sca-Gal4 tub-Gal80*$^{ts}$*/+ ; UAS-IKKε::myc spnF*$^{1}$*/spnF*$^{1}$ | 2H, S2D |
| *w; neu-Gal4 UAS-Spn-F::GFP/TM6B* | 2L, 3H, 5A |
| *w;; neu-Gal4 tub-Gal80*$^{ts}$ */UAS-Dhc64c*$^{RNAi}$ *UAS-Spn-F::GFP* | 2M |
| *w;; neu-Gal4 tub-Gal80*$^{ts}$*/UAS-Dhc64c*$^{RNAi}$ | 2O |
| *w; Sca-Gal4/UAS-HA::DHC* | 2R |
| *w; ScaGal4 IR2615R/UAS-HA::DHC* | 2S |
| *w; Sca-Gal4/IR2615R; spnF*$^{1}$*/spnF*$^{1}$ | 3E |
| *w; Sca-Gal4/+; UAS-Spn-F::GFP spnF*$^{1}$*/spnF*$^{1}$ | 3F, 5F |
| *w; Sca-Gal4 tub-Gal80*$^{ts}$*/+; UAS-IKKε::myc spnF*$^{1}$*/ spnF*$^{1}$ | 3G |
| *y w Ubx-flp/w; ikkε*$^{66}$ *FRT40A/ubi-GFP FRT40A* | 3I |
| *y w Ubx-flp/w; ikkε*$^{66}$ *FRT40A/tub-Gal80 FRT40A ; UAS-Spn-F::GFP/da-Gal4 UAS-mKO* | 3J |
| *y w Ubx-flp/w; ikkε*$^{66}$ *FRT40A/tub-Gal80 FRT40A ; UAS-IKKε::myc/da-Gal4 UAS-mKO* | 3K |

| | |
|---|---|
| *w; Sca-Gal4 tub-Gal80*$^{ts}$*/+; UAS-IKKε::myc/+* | 3L |

| genotype | Figure numbers |
|---|---|
| *w; Sca-Gal4/UAS-ΔC2::GFP* | 5B, S4D |
| *w; Sca-Gal4/UAS-Δ212::GFP* | 5C, S4G |
| *w; Sca-Gal4/UAS-Δ253::GFP* | 5D, S4H |
| *w; Sca-Gal4/UAS-ΔC2::GFP; spnF*$^{1}$*/spnF*$^{1}$ | 5G, S4D’ |
| *w; Sca-Gal4/UAS-Δ212::GFP; spnF*$^{1}$*/spnF*$^{1}$ | 5H, S4G’ |
| *w; Sca-Gal4/UAS-Δ253::GFP; spnF*$^{1}$*/spnF*$^{1}$ | 5I, S4H’ |
| *w;; jvl*$^{1}$*/jvl*$^{1}$ | 6A, 6C, 6E, 6G, 6I |
| *w; Sca-Gal4/+; UAS-Jvl::GFP/+* | 6L, 6N |
| *w; Sca-Gal4 IR2615R/+; UAS-Jvl::GFP/+* | 6M, 6O |
| *y w Ubx-flp/w; ikkε*$^{alice}$ *FRT40A/ubi-GFP FRT40A* | S2D |
| *y w Ubx-flp/w; ikkε*$^{alice}$ *FRT40A/tub-Gal80 FRT40A ; UAS-Spn-F::GFP/da-Gal4 UAS-mKO* | S2E |
| *w; Sca-Gal4/UAS-N::GFP* | S4A |
| *w; Sca-Gal4/UAS-C::GFP* | S4B |
| *w; Sca-Gal4/UAS-ΔC1::GFP* | S4C |
| *w; Sca-Gal4/UAS-C1::GFP* | S4E |
| *w; Sca-Gal4/UAS-C2::GFP* | S4F |
| *w; Sca-Gal4/UAS-N::GFP; spnF*$^{1}$*/spnF*$^{1}$ | S4A’ |
| *w; Sca-Gal4/UAS-C::GFP; spnF*$^{1}$*/spnF*$^{1}$ | S4B’ |
| *w; Sca-Gal4/UAS-ΔC1::GFP; spnF*$^{1}$*/spnF*$^{1}$ | S4C’ |
| *w; Sca-Gal4/UAS-C1::GFP; spnF*$^{1}$*/spnF*$^{1}$ | S4E’ |
| *w; Sca-Gal4/UAS-C2::GFP; spnF*$^{1}$*/spnF*$^{1}$ | S4F’ |

**Table S2. Antibodies and detection reagents used in this study**

| Antibody | Source | Dilution |
|---|---|---|
| Mouse anti-Spn-F (8C10) | Abdu et al., 2006 | 1/2 for WB |
| Rabbit anti-Spn-F | This study | 1/100 for IF/IHC |
| | | 1/500 for IP |
| Guniea pig anti-Spn-F | This study | 1/100 for IHC |
| Mouse anti-IKKε (#80) | Oshima et al., 2006 | 1/20 for WB |
| Rabbit anti-pIKKε (S175) | Otani et al., 2011 | 1/200 for IHC |
| Mouse anti-DHC (2C11-2) | DSHB (Sharp et al., 2000) | 1/5 for WB |
| Mouse anti-α-tubulin (DM1A) | SIGMA (T9026) | 1/500 for IF |
| Rabbit anti-GFP | MBL (598) | 1/200 for IHC |
| | | 1/300 for IP |
| Rabbit anti-myc | Santa Cruz (sc-789) | 1/100 for IHC |
| | | 1/500 for WB |
| Mouse anti-HA (16B12) | BioLegend (901513) | 1/2,000 for IHC |
| | | 1/5,000 for WB |
| Rabbit anti-GFP HRP-DirecT | MBL (598-7) | 1/10,000 for WB |
| Mouse anti-myc HRP-DirecT | MBL (M047-7) | 1/5,000 for WB |
| Rabbit anti-HA HRP-DirecT | MBL (561-7) | 1/5,000 for WB |
| Goat anti-Mouse IgG Alexa-488 | Molecular Probes (A-11029) | 1/200 for IHC |
| Goat anti-Mouse IgG Alexa-568 | Molecular Probes (A-11004) | 1/200 for IF/IHC |
| Goat anti-Mouse IgG Cy5 | Amersham (PA45010) | 1/100 for IHC |
| Goat anti-Rabbit IgG Alexa-488 | Molecular Probes (A-11034) | 1/200 for IHC |
| Goat anti-Rabbit IgG Alexa-568 | Molecular Probes (A-11036) | 1/200 for IHC |
| Goat anti-Rabbit IgG Cy5 | Amersham (PA45011) | 1/100 for IHC |

| Antibody | Source | Dilution |
|---|---|---|
| Goat anti-Guinea pig Cy3 | Chemicon (AP108C) | 1/200 for IHC |
| Alexa-488 conjugated phalloidin | Molecular Probes (A-12379) | 1/50 for IHC |
| Alexa-568 conjugated phalloidin | Molecular Probes (A-12380) | 1/50 for IHC |
